# Supplementary material for: Probing boron vacancy defects in hBN via single spin relaxometry
Source: Nat Commun. 2026 Mar 10;17:3718. doi: 10.1038/s41467-026-70545-6 (PMC13103406; doi:10.1038/s41467-026-70545-6)
Supplement: Supplementary file 1 — Supplementary Information [file 41467_2026_70545_MOESM1_ESM.pdf]

Supplementary Information for
Probing Boron Vacancy Defects in hBN via Single Spin Relaxometry

Alex L. Melendez [1], Ruotian Gong [2], Guanghui He [2], Yan Wang [3], Yueh-Chun Wu [4], Thomas Poirier [5], Steven Randolph [1], Sujoy Ghosh [1], Liangbo Liang [1], Stephen Jesse [1], An-Ping Li [1], Joshua T. Damron [6], Benjamin J. Lawrie [4], James H. Edgar [5], Ivan V. Vlassiouk [1], Chong Zu [2], Huan Zhao [1*]

[1]Center for Nanophase Materials Sciences, Oak Ridge National Laboratory, Oak Ridge, TN 37831, USA.

[2]Department of Physics, Washington University in St. Louis, St. Louis, MO 63130, USA.

[3]Computational Sciences and Engineering Division, Oak Ridge National Laboratory, Oak Ridge, TN 37831, USA.

[4]Materials Science and Technology Division, Oak Ridge National Laboratory, Oak Ridge, TN 37831, USA.

[5]Tim Taylor Department of Chemical Engineering, Kansas State University, Manhattan, KS 66506, USA.

[6]Chemical Sciences Division, Oak Ridge National Laboratory, Oak Ridge, TN 37831, USA.

[*]Corresponding author. Email: zhaoh1@ornl.gov

This PDF file includes:

Supplementary Text

Figs. S1 to S27

Table S1

Contents

Supplementary Note 1 NV-Sample Distance Estimation S2

Supplementary Note 2 Disentangling PL Signals from Optically Active Spin Defects S8

Supplementary Note 3 Optimization and Sensitivity of T_1-MR Measurements S13

Supplementary Note 4 Derivation of NV Relaxation Rate from Spin Noise in hBN S22

Supplementary Note 5 ODMR of V_B^- in $h^{10}B^{15}N$ S32

Supplementary Note 6 Simulation of He Ion Irradiation in hBN/Au S36

Supplementary Note 7 Additional T_1-MR Measurements S38

Supplementary Note 8 Gate-tunable Modulation of Near-Surface V_B^- Density S40

Supplementary Note 9 Additional Imaging of hBN_{nat} Sample S42

Supplementary Note 1 NV-Sample Distance Estimation

Achieving a short NV-sample distance is essential for enabling measurable cross-relaxation between the NV center and the V_B^- ensemble in hBN. This distance is influenced by four key factors:

1. the implantation depth of the NV center,
2. the tip-sample mechanical standoff distance,
3. the smoothness and cleanliness of the NV tip surface, and
4. the surface condition of the hBN sample.

In this work, NV centers were created by implanting nitrogen ions at 6 keV, yielding a mean implantation depth of 9 ± 4 nm, as simulated using SRIM. For these 6 keV-implanted NV centers, the measured longitudinal relaxation times T_1 typically fall in the 2-8 ms range.A typical PL

counts used during ODMR measurement is 200-500 kcts/s, and a typical CW-ODMR sensitivity is $2-3\,\mu\mathrm{T}/\sqrt{\mathrm{Hz}}$.

For cross-relaxometry measurements, we screened more than ten newly prepared NV probes and selected three new tips exhibiting both shorter T_1 values—often indicative of shallower effective NV depth—and flat tip surfaces, which are critical for achieving a small NV-sample separation. We also used an old tip with a flat surface, which is the "short T_1 tip" used in Figure 2e (Probe #3). For the new tips (Probes #1, 2 and 4), we intentionally selected probes with raw T_1 values between 1 and 5 ms (see Table S1). We also tested an ultra-shallow NV probe fabricated using 3 keV implantation. Although this probe exhibited clear cross-relaxation signatures, its low photoluminescence brightness and reduced ODMR contrast limited sensitivity, and it was therefore not used for data acquisition in this study. The tip-sample distance was regulated using a frequency-modulated feedback mechanism, where the tuning fork was driven at resonance and an off-resonance frequency shift was held at a constant setpoint. A shift of 20 ± 5 Hz was used to ensure tight contact during scanning.

According to a technical note by Qnami using the same hardware and probe type, a frequency shift of 15 Hz corresponds to a tip-sample distance of < 5 nm. When combined with a 9 nm implantation depth, this yields an NV-sample distance of < 14 nm [Qnami, *Antiferromagnetic Spin Cycloids in BiFeO*$_3$].

To further ensure optimal NV-sample contact, we developed a PL-assisted feedback method based on the boron-vacancy (V_B^-) emission collected through the nanopillar waveguide of the NV probe. During these measurements, a 750 nm long-pass filter was inserted in the detection path so that the collected signal predominantly originated from V_B^-. As the tuning-fork frequency offset (used as the feedback setpoint) was increased under small setpoint conditions ($<$10 Hz), the V_B^- PL intensity rose sharply. With further increases in the setpoint, the PL intensity increased more gradually and eventually saturated, typically at 20-40 Hz (Fig. S1). This saturation behavior indicates a stable NV-sample separation, beyond which further driving of the tuning fork does not improve contact. We use this saturation as an operational criterion for establishing optimal contact, which is ultimately corroborated by the observation of strong cross-relaxation signals.

To maintain probe cleanliness and surface quality, we used either brand-new NV probes or cleaned previously used probes (here, "used" refers only to probes employed within the same cross-relaxometry experiment). Cleaning was performed using an NT-MDT TGT1 test grating,

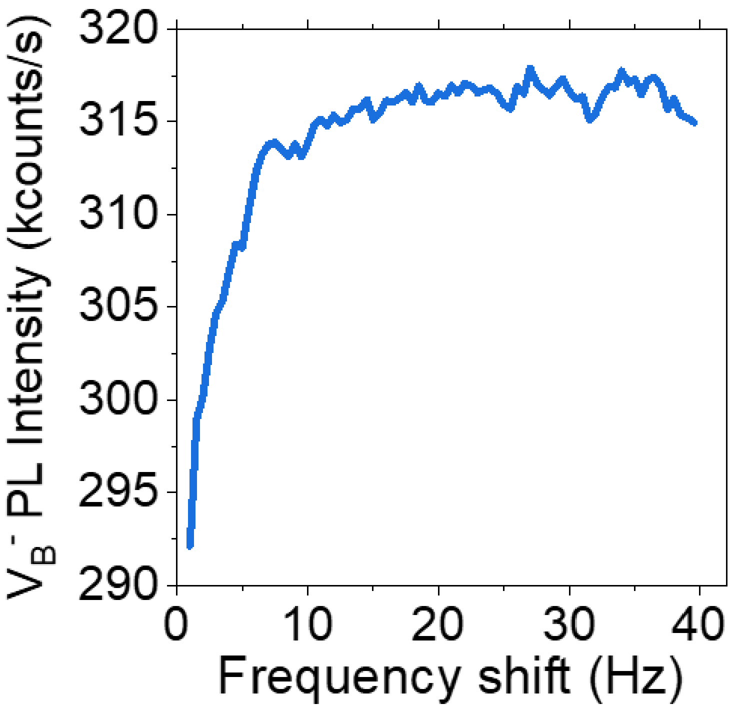

Fig. S1: **Determination of Optimal NV-sample Contact:** The V_B^- fluorescence signal collected using the scanning NV tip when adjusting the off-resonance frequency of the tuning fork. A 750 nm long-pass filter was applied to block NV emissions.

which consists of a dense array of sharp needles. This grating also enabled reverse AFM imaging: by scanning the tip across a single sharp needle with a small step size, we obtained the surface profile of the tip itself. Figure S2a displays a contaminated tip characterized by surface bumps, while Figure S2b shows the same tip after cleaning, revealing a smooth and well-defined profile. In practice, we first use reverse imaging to identify suitable flat probes, and after each experiment we reevaluate the tip surface and perform additional cleaning via ∼10 min UV-ozone treatment and scanning of the TGT1 grating. We note that the NV T_1 can vary slightly after each measurement-cleaning cycle. In addition, unlike many previous studies that employ tapered NV probes, we use truncated-paraboloid probes with flat end facets, which significantly reduce the minimum achievable NV-sample separation.

Before relaxometry measurements, the CVD-grown hBN was annealed at 350°C to remove surface contaminants. BN is generally believed to be one of the smoothest surfaces, which helps minimize tip-sample distance. Throughout the experiments, we observed that scanning the hBN surface caused significantly less tip contamination than scanning cleanroom-fabricated CoFeB

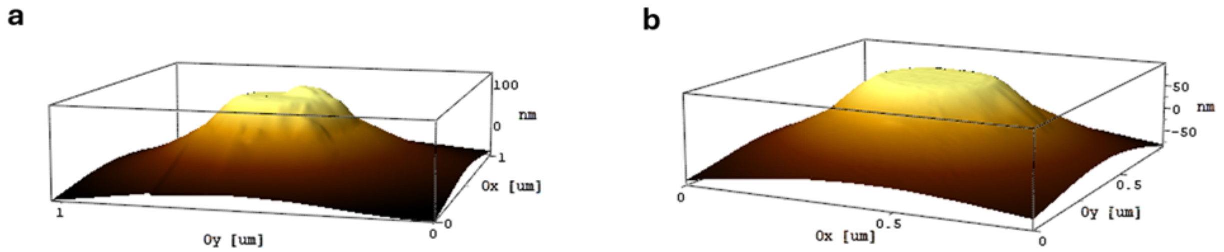

Fig. S2: **NV Tip Surface Profile:** Surface profile of NV tip obtained through scanning the tip across a sharp needle (a) before and (b) after tip cleaning.

samples, indicating the high surface cleanliness of our hBN.

To determine our NV-sample distance more accurately, we scanned a patterned magnetic multilayer stack (Ta 2.5 nm / CoFeB 2 nm / Ta 2.5 nm) structured into 1 μm-wide magnetic stripes (Fig. S3a,b). We first measured the NV-sample distance of the NV tip that was used to obtain data for main manuscript Fig. 1, Fig. 2d, and Figure 3. We refer to this as Probe #1. Scanning was performed under a 40 G out-of-plane magnetic bias, using a 20 Hz frequency shift setpoint. The magnetic field from the stripe was modeled using Maxwell's equations (*1*), revealing that the linewidth of the magnetic field profile near the stripe edge is approximately $2d$, where d is the NV-sample distance and the peak field scales as $1/d$. This technique has been demonstrated by previous studies (*2*).

We adopted a lift-mode scanning strategy to avoid issues associated with full-contact scanning, as the latter requires smaller step sizes to resolve the sharp field gradients but often leads to tip contamination and increased standoff distances. Moreover, in full contact, the strong magnetic field from CoFeB can severely quench the NV ODMR signal.

In the lift-mode scans, we first performed a rough AFM topography scan with 100 nm steps, then lifted the probe by 80 nm and performed an ODMR line scan with 18 nm steps (Fig. S3c). A second line scan was conducted at 120 nm lift height. Using the extracted field profiles and applying the $B \sim 1/d$ scaling at both edges of the stripe, we determined the NV-sample distance to be 13.9 ± 1.5 nm after subtraction the tip lift height. After further subtracting the 2.5 nm capping layer thickness, the final NV-to-sample surface distance is 11.4 ± 1.5 nm.

As an independent estimate of the NV-sample distance, we also analyzed the linewidths of magnetic features measured in the 80 nm lift-height scan, fitting the data using the model described

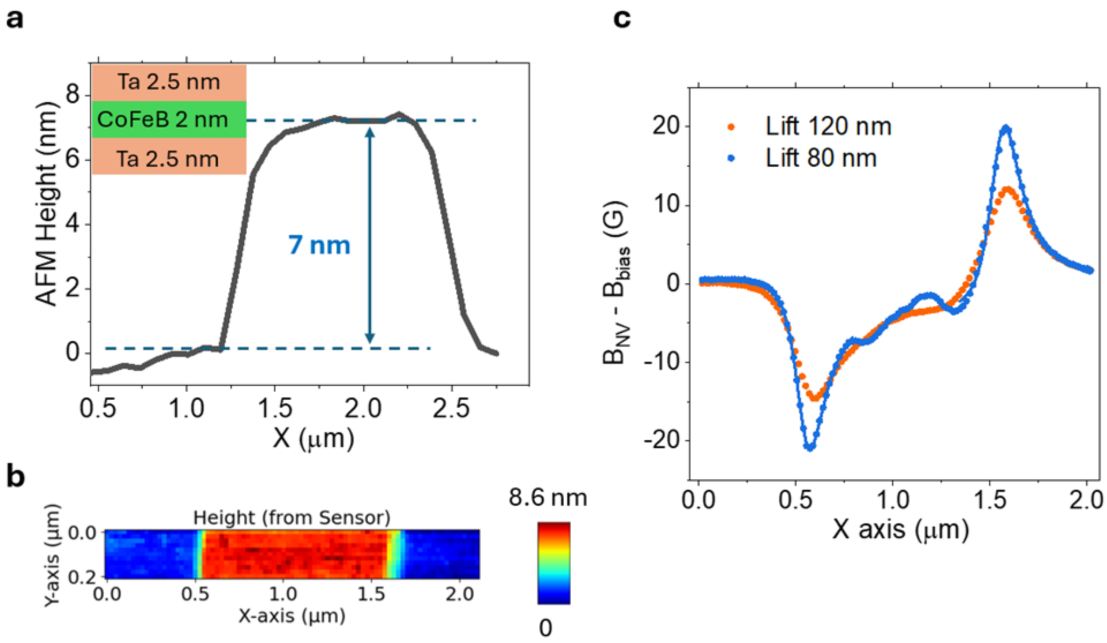

Fig. S3: **Measuring NV-Sample Distance:** (a) Height profile and (b) AFM topography map of the patterned Ta/CoFeB/Ta magnetic stripe structure. (c) Magnetic field measured at the NV center location after subtraction of the applied out-of-plane bias field used to pre-split the NV ODMR spectrum. Line scans were performed at tip lift heights of 80 nm and 120 nm, respectively. The two peaks of the linecut of 80 nm lift height are fitted using the equations in Supplementary Information (*2*) (dot: data; line: fitted curve).

in (*2*). From the left magnetic peak, we obtained an apparent distance of 94.5±0.5 nm, corresponding to 12.0 ± 0.5 nm after accounting for the 80 nm lift height and the capping layer. Fitting the right peak yielded 13.8 ± 2.6 nm, giving an average NV-sample distance of 12.9 ± 1.9 nm. Because this linewidth-based approach is sensitive to probe geometry and tends to overestimate the true separation (*3*), we do not rely on it as our primary distance estimate.

We also independently determined the NV-sample distance for Probe #4, used for the data shown in Figs. S17 and S25-S26. In this case, we scanned the probe over the same CoFeB nanomagnet at different lift heights and extracted the maximum stray field $B_{\max}$ as a function of lift height h. A linear fit of $1/B_{\max}$ versus h yields the slope and intercept, from which the NV-sample distance can be obtained as intercept/slope. This analysis yields an NV-sample distance of 19.25 ± 2.03 nm (Fig. S4), consistent with an independent estimate obtained from a purely cross-relaxometry–based method (Fig. S17), validating our distance determination.

For comparison, we applied the same lift-mode method to an older probe with a degraded surface profile (Fig. S5a). The extracted NV-sample distance for this probe was 48.18 ± 2.14 nm

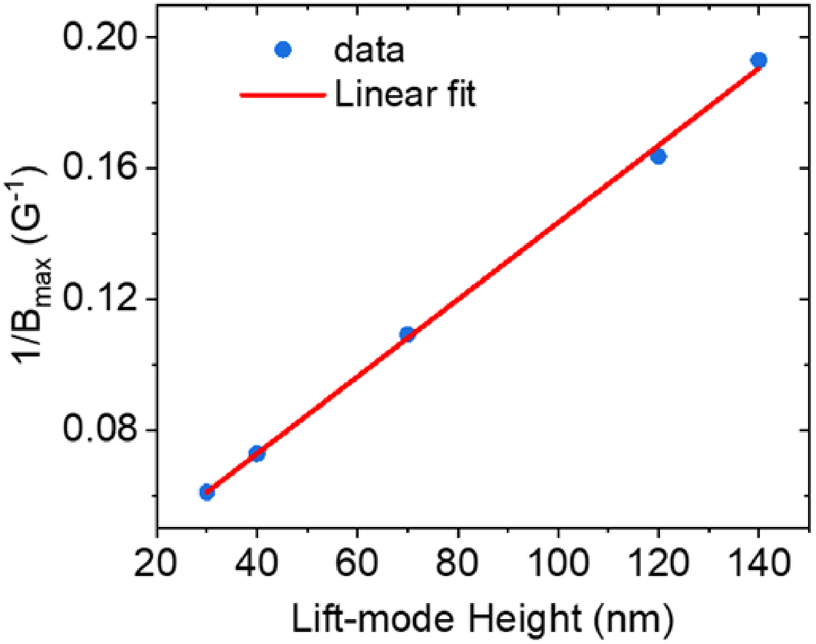

Fig. S4: **Determination of NV-sample Distance Using Inverse Field Dependence:** Tip lift height dependent maximal measured stray field when scanning an NV tip (Probe #4) across the same magnetic stripe in Figure S3. The $1/B_{max}$ vs lift height curve was linearly fitted to extract an initial NV-sample distance of 19.25 ± 2.03 nm.

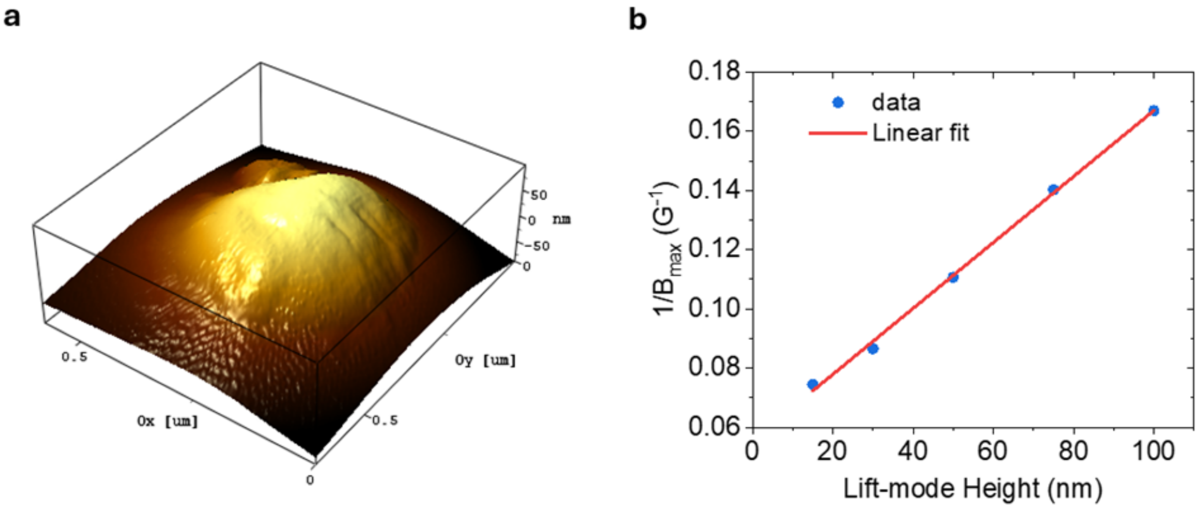

Fig. S5: **NV-Sample Distance of an Used Tip with Degraded Surface Quality:** (a) The surface profile of the used tip obtained using the reverse imaging technique. (b) Tip lift height dependent maximal measured stray field when scanning the old NV tip across the same magnetic stripe in Figure S3. The $1/B_{max}$ vs lift height curve was linearly fitted to extract an initial NV-sample distance of 48.18 ± 2.14 nm.

Probe #	1	2	3	4
Figures	1c-d, 2d, 3f, S2b, S3, S8, S23b	2b-c, S21, S23a	2e-f, S24	S4, S17, S25-26
T_1 at non-CR (tip engaged) [ms]	1.34 (new tip), 1.21 (after cleaning)	2.09 (new tip w/ flat surface)	~0.1 (used tip w/ flat surface)	5.19 (new tip w/ flat surface)
T_1 at CR [ms]	0.406 (on hBN_{nat})	1.21 (on $h^{10}B^{15}N$)	0.053 (on $h^{10}B^{15}N$)	3.49 (on $h^{10}B^{15}N$), 1.19 (on hBN_{nat})
NV-sample distance [nm]	11.4 ± 1.5 (magnet-lift mode), 12.9 ± 1.9 (magnet scan)	N/A	N/A	19.25 ± 2.03 (magnet scan), 21.1 ± 5 (T_1 technique)

Table S1: A list of NV probes used to obtain the experiment data in this work.

(Fig. S5b), highlighting the critical importance of a clean, flat probe surface for achieving minimal NV-sample separation and in turn a high cross-relaxometry sensitivity. This old tip is not used for data acquisition in cross-relaxometry studies in this work.

Table S1 summarizes the NV probes used in this work and their key properties. All probes were fabricated using nitrogen implantation at an energy of 6 keV. Probe #3 is the only previously used probe; despite its reduced T_1, it maintains a flat tip surface and therefore remains suitable for measurements.

Supplementary Note 2 Disentangling PL Signals from Optically Active Spin Defects

While we have proposed a method to probe a wide variety of spin-active quantum defects, there are cases such as that of using an NV to sense V_B^- spins when both the defect and the sensor emit PL. In this case, one must seek a method to differentiate changes in the PL of the sensor from that of the defect. Using a pulsed T_1 relaxometry protocol, there are three ways in which PL between the two can be distinguished. If there is a large enough difference of the emission wavelength, then the defect PL can be blocked by optical filtering while allowing PL from the sensor to be collected. In this work, a 750 nm shortpass filter blocked the majority of the PL from the V_B^- centers when all measurements of the NV PL were performed (Fig. S6). Figure S7 and shows a spatial scan of the

hBN_{nat} sample comparing unfiltered versus filtered V_B^- PL. The PL intensity decreased by an order of magnitude when filtered, allowing changes in the NV PL to be seen more clearly. Figure S8 shows ODMR of the single NV and V_B^- ensemble in hBN_{nat}. The V_B^- ODMR was measured with the NV and shortpass filter absent, while the NV ODMR was measured before and after engaging with the sample surface including the optical filtering. No discernible PL signal at the V_B^- resonance frequency is observed in the NV ODMR.

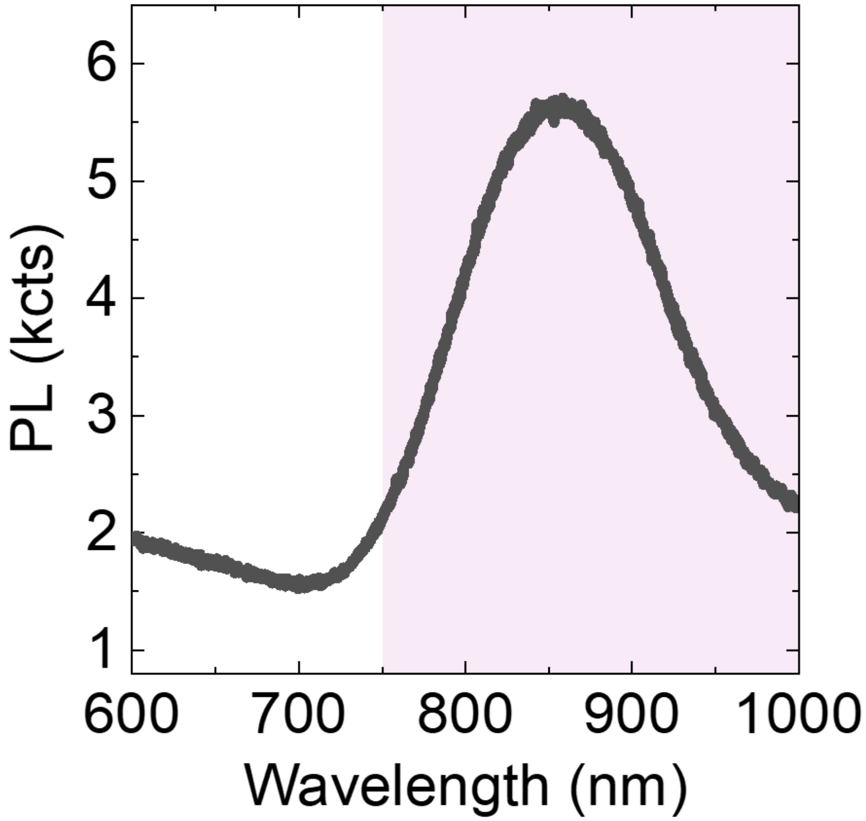

Fig. S6: **Boron vacancy center photoluminescence spectrum:** PL of hBN_{nat} sample irradiated with He ions as a function of wavelength. A peak near 850 nm is consistent with the expected PL spectrum of V_B^- centers. Placement of a 750 nm shortpass filter will block most of the V_B^- PL as shown by the pink shaded region.

In addition to differences between spin defect PL spectra, changes in the PL can be further separated if there is a difference between their radiative lifetimes. This method is also employed in our measurements, as depicted in Fig. S9. A laser pulse excites the NV in order to read out its PL; however, this also excites nearby V_B^- centers which emit PL as well. Given the shorter radiative lifetime for V_B^- centers of $\tau_{V_B^-} = 1.6$ ns compared to that for the NV of $\tau_{NV} = 12$ ns, then introduction of a 6 ns delay time before the photon counting window is opened allows for most of

the V_B^- PL to disappear before a measurement is made. Thus, even if both defects emit PL at the same wavelength precluding optical filtering, the PL signals can still be separated by engineering the counting window to take advantage of the difference in radiative lifetime.

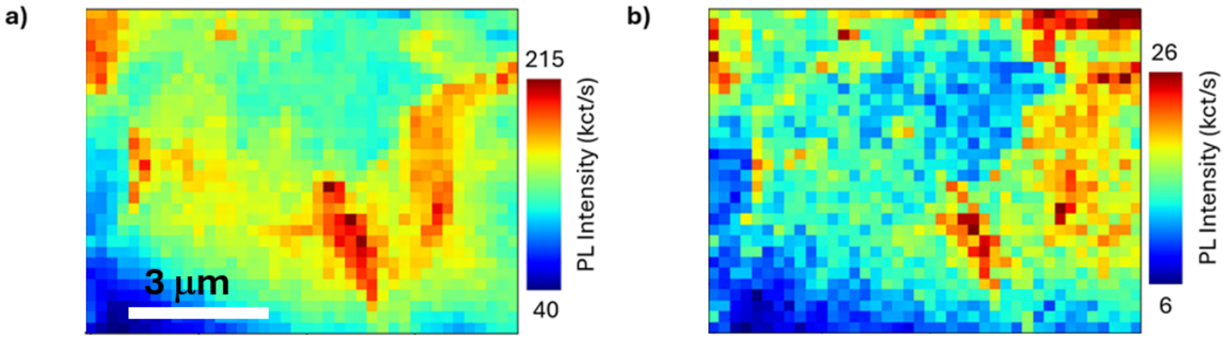

Fig. S7: **Spatial map of boron vacancy center photoluminescence in hBN$_{\mathbf{nat}}$:** (a) V_B^- PL as a function of position without optical filtering. (b) V_B^- PL with 750 nm shortpass filter showing an order of magnitude decrease in PL, allowing changes in the NV PL to be detected independently of changes in the V_B^- PL. Under the same measurement conditions, our NV center has PL intensity of approximately 600 kcts/s (without filtering) and 500 kcts/s (with filtering)

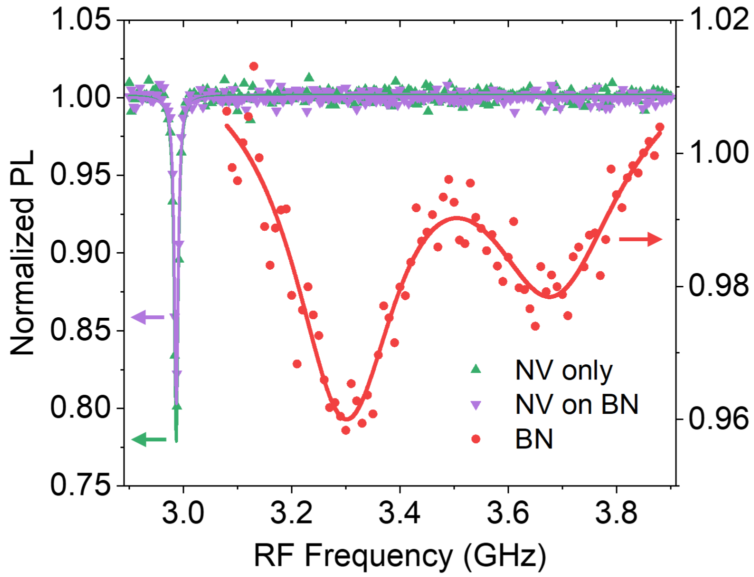

Fig. S8: **Independent ODMR Spectra:** CW-ODMR spectra of the NV center before (green curve) and after (purple curve) engaging with the hBN$_{\text{nat}}$ sample, and of the V_B^- centers (red curve), measured under an out-of-plane magnetic field of 68.9 G. All spectra are fitted using Lorentzian functions. The left and right vertical axes correspond to the NV and V_B^- data, respectively. In the NV ODMR curve, only the $m_s = 0 \leftrightarrow +1$ transition is plotted.

Lastly, the PL signals can be differentiated through differences in the longitudinal relaxation

times T_1. The longitudinal relaxation time of the NV here ranges from 100s of μs to a few ms, while that of the V_B^- centers is on the order of 1s to 10s of μs as shown in Fig. S10. Because of this, the PL as a function of the evolution time τ is only affected by the relaxation of the V_B^- centers for roughly the first 10s of μs. After that, the PL decay curve is entirely determined by the NV relaxation. Thus, the NV T_1 can be determined simply by omitting the first 50 μs of PL data in the curve fitting or, in the case of iso-T_1 measurements, by adding a delay before the reference PL readout is taken. In this way, NV relaxation can still be measured independently of V_B^- relaxation, even if optical filtering and radiative-lifetime differences cannot be exploited. In this work, all three methods are used to ensure that changes in V_B^- PL do not confound measurements of the NV relaxation rate or introduce a substantial static background PL.

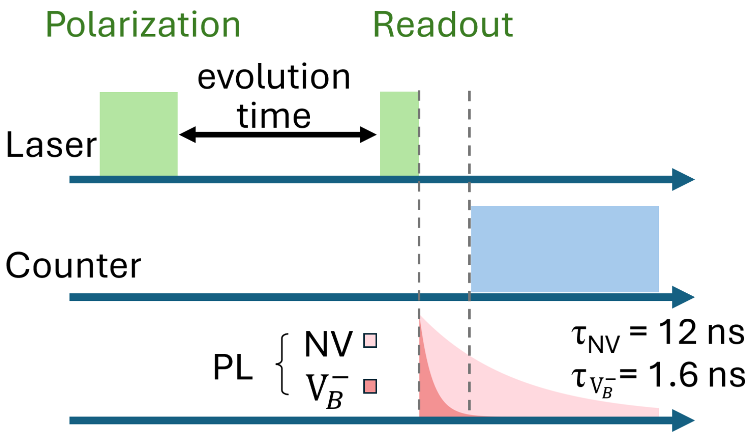

Fig. S9: **Pulse sequence used for T_1-MR measurements:** Each cycle begins with green laser excitation to polarize the NV center into the $m_s = 0$ state, followed by a free evolution time τ. A time-gated dark readout scheme is then implemented: a 10 ns laser pulse excites the NV, and a photon counter is activated 6 ns after the laser turns off, collecting fluorescence for 30 ns. This “10 ns laser + 30 ns counter” sequence is repeated 15 times within each cycle to accumulate signal. After re-polarizing the NV to spin zero state, a reference signal is then acquired using an additional “10 ns laser + 30 ns counter” sequence. Simulated photoluminescence decay curves for NV and V_B^- centers are shown to illustrate that, under this timing scheme, only NV fluorescence is collected.

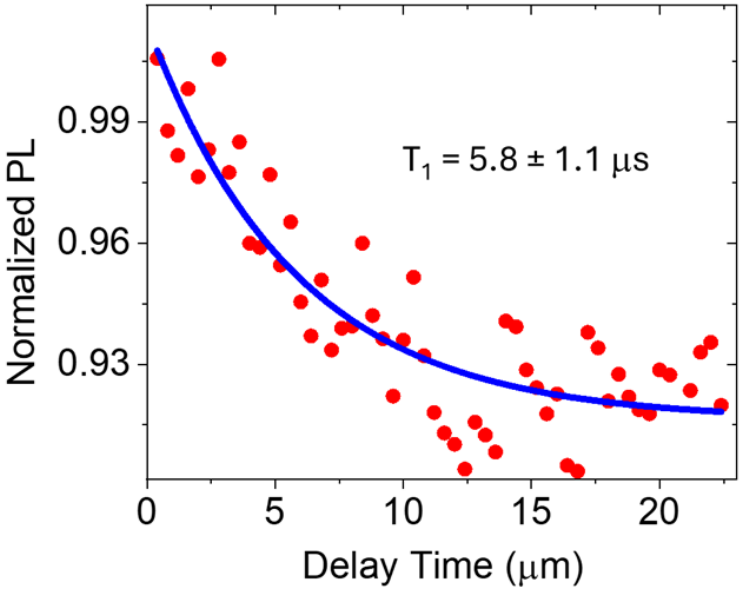

Fig. S10: **Relaxation time of boron vacancy centers in hBN:** Normalized PL as a function of delay time showing relaxation of V_B^- spins to equilibrium.

Supplementary Note 3 Optimization and Sensitivity of T_1-MR Measurements

Excluding noise, the optimal τ value to achieve maximum contrast in a single-τ T_1-MR measurement can be found by considering the difference between two PL decay curves of the form:

$$n(\tau;\, T_1) = I_0\, t_{\mathrm{ro}}\left[(1 - C) + Ce^{-\tau/T_1}\right]. \tag{S1}$$

Here $n(\tau;\, T_1)$ denotes the mean number of detected photons (counts) acquired in a fixed optical readout window at the end of a single measurement cycle after a wait time τ. The parameter I_0 is the detected photon count rate (counts/s) during the readout window at $\tau = 0$, t_{ro} is the total readout duration per cycle, and C is the PL contrast.

Let T_1 be the background relaxation time far from the resonance/CR condition, T_1^{CR} be the relaxation time at the resonance/CR condition, and

$$r = \frac{T_1^{\mathrm{CR}}}{T_1} < 1 \tag{S2}$$

be their ratio. The background PL decay curve is then given by $n_{\mathrm{bg}}(\tau) \equiv n(\tau;\, T_1)$ and the resonant/CR curve is $n_{\mathrm{CR}}(\tau) \equiv n(\tau;\, rT_1)$. The (noise-free) contrast between these two signals is then

$$\delta n(\tau) \equiv n_{\mathrm{bg}}(\tau) - n_{\mathrm{CR}}(\tau) = CI_0 t_{\mathrm{ro}}\left[e^{-\tau/T_1} - e^{-\tau/rT_1}\right]. \tag{S3}$$

The value of τ that maximizes the difference $\delta n(\tau)$ is easily found to be

$$\tau = -T_1 \frac{r}{1-r} \log r\,. \tag{S4}$$

As expected the optimal choice of τ depends on r, i.e., how much the relaxation time changes on vs off resonance. This dependence is shown in the inset of Fig. S11.

However, in a real experiment the shot noise of photon counts scales as the square root, and a shorter choice of τ allows more measurement cycles to be completed within a given total amount

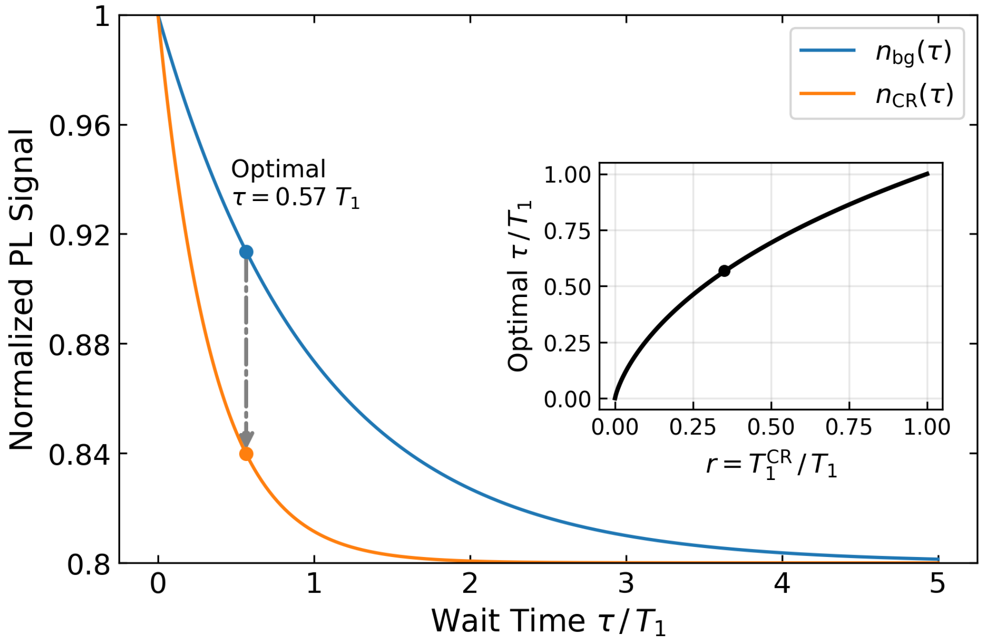

Fig. S11: **Optimization of wait time τ for single-τ T_1-MR Measurements:** Theoretical PL decay curves showing optimal choice of wait time τ that maximizes their difference for a given r. The blue curve $n_{bg}(\tau)$ is the PL decay curve with the off-resonance (i.e., background) T_1, while the orange curve $n_{CR}(\tau)$ is the PL decay at the CR condition. The optimal τ determines the location of the gray dashed line. Plotted with $C = 0.2$, $r = 0.35$ and $I_0 t_{ro} = 1$. Inset: Optimal τ as a function of r from Eq. (S4), with the point located at $\tau = 0.57\,T_1$.

of measurement time T_m. Let a single measurement cycle have total duration

$$t_{cyc} = \tau + t_{dead}, \tag{S5}$$

where t_{dead} accounts for all overhead time per cycle (initialization, readout, and any additional dead time). The number of cycles is then approximately $m \simeq T_m/t_{cyc}$. The total number of detected counts accumulated at a wait time τ during a total measurement time T_m is therefore

$$N(\tau;\,T_1) = I_0 t_{ro} \frac{T_m}{t_{cyc}} \left[(1-C) + Ce^{-\tau/T_1}\right]. \tag{S6}$$

For later convenience we define the corresponding mean total counts under the background and CR conditions as $N_{bg}(\tau) \equiv N(\tau;\,T_1)$ and $N_{CR}(\tau) \equiv N(\tau;\,rT_1)$. We also define the signal amplitude associated with switching the relaxation time from T_1 to rT_1 as the change in the expectation value

of the measured observable N:

$$\delta N(\tau) \equiv N_{\rm bg}(\tau) - N_{\rm CR}(\tau) = C I_0 t_{\rm ro} \frac{T_m}{t_{\rm cyc}} \left[e^{-\tau/T_1} - e^{-\tau/rT_1} \right]. \tag{S7}$$

Importantly, $\delta N(\tau)$ here is not the result of subtracting two independently noisy measurements. Rather, it is the predicted change in the mean value of a single measured quantity N when the underlying relaxation time changes from T_1 to rT_1. In the shot-noise-limited regime, the measured total photon number N is well approximated as Poisson-distributed with variance equal to its mean, so that $\sigma_N \simeq \sqrt{N}$.

If the background expectation $N_{\rm bg}(\tau)$ is known and one performs a measurement under the CR condition, then a natural definition of the single-measurement SNR is

$$\mathrm{SNR}(\tau) = \frac{|\delta N(\tau)|}{\sigma_N} \simeq \frac{\left|N_{\rm bg}(\tau) - N_{\rm CR}(\tau)\right|}{\sqrt{N_{\rm CR}(\tau)}}. \tag{S8}$$

Using Eq. (S6) this yields (Fig. S12)

$$\mathrm{SNR}(\tau) = C \sqrt{I_0 t_{\rm ro} \frac{T_m}{t_{\rm cyc}}} \, \frac{e^{-\tau/T_1} - e^{-\tau/rT_1}}{\sqrt{(1-C) + C e^{-\tau/rT_1}}}. \tag{S9}$$

The value τ that maximizes the SNR is obtained by solving $\frac{\partial}{\partial \tau} \ln\left[\mathrm{SNR}(\tau)\right] = 0$, which gives the transcendental condition

$$\frac{-\frac{1}{T_1} e^{-\tau/T_1} + \frac{1}{rT_1} e^{-\tau/rT_1}}{e^{-\tau/T_1} - e^{-\tau/rT_1}} + \frac{1}{2} \frac{\frac{C}{rT_1} e^{-\tau/rT_1}}{(1-C) + C e^{-\tau/rT_1}} = \frac{1}{2} \frac{1}{t_{\rm cyc}} \frac{\partial t_{\rm cyc}}{\partial \tau}. \tag{S10}$$

For the simple case of constant overhead $t_{\rm cyc} = \tau + t_{\rm dead}$ one has $\frac{\partial t_{\rm cyc}}{\partial \tau} = 1$, and Eq. (S10) can be solved numerically for τ. In the further limit $t_{\rm dead} \to 0$, Eq. (S10) reduces to the $t_{\rm cyc} \approx \tau$ optimization condition used to generate the inset of Fig. S12.

To determine the magnetic sensitivity of this measurement, we now allow the relaxation rate to depend on magnetic field. It is convenient to define the relaxation rates $\Gamma_1 \equiv 1/T_1$ and $\Gamma_1^{\rm CR} \equiv 1/T_1^{\rm CR}$,

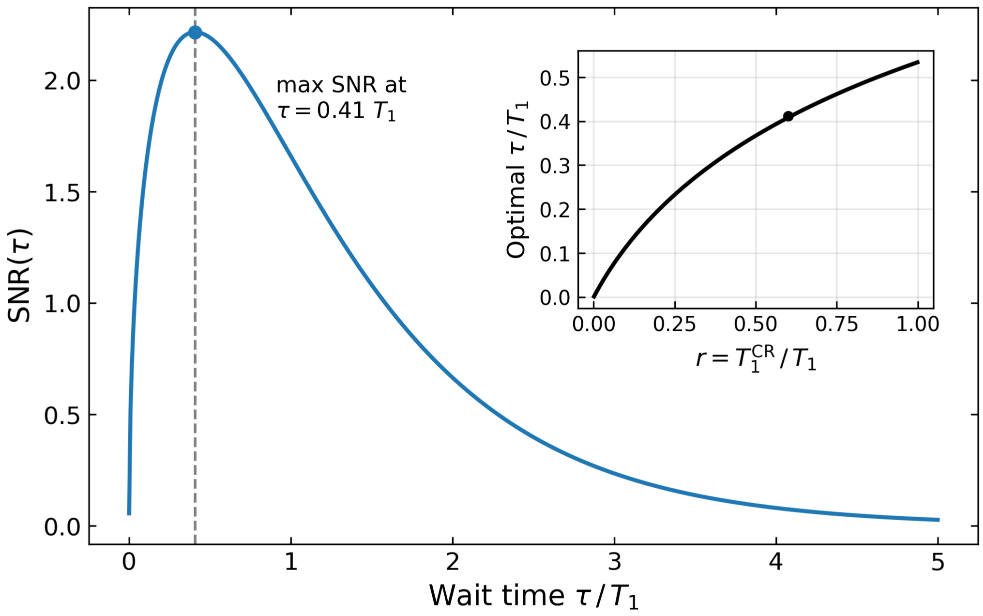

Fig. S12: **Optimization of T_1-MR SNR as a function of τ:** SNR of single-τ T_1-MR measurement from Eq. (S9). The maximum occurs at the optimal τ. Plotted with $C = 0.2$, $I_0 t_{ro} = 0.18$ and $T_m = 10^4\, T_1$, assuming $t_{cyc} \approx \tau$. Inset: Optimal τ as a function of r numerically solved in the $t_{cyc} \approx \tau$ limit, with the point located at $\tau = 0.41\, T_1$.

so that the ratio can be written equivalently as

$$r = \frac{T_1^{CR}}{T_1} = \frac{\Gamma_1}{\Gamma_1^{CR}}\,. \tag{S11}$$

Instead of using a fixed relaxation rate, consider a Lorentzian magnetic field dependence of the form

$$\Gamma_L(B) = \Gamma_1 \left[1 - \frac{(1 - 1/r)\lambda^2}{(\omega - \omega_{CR})^2 + \lambda^2} \right], \tag{S12}$$

for a given HWHM λ. Here $\omega \equiv \omega(B)$ and $\omega_{CR} \equiv \omega(B_{CR})$ where B_{CR} is the field at which the center frequencies of the NV $m_s = 0 \leftrightarrow +1$ and V_B^- $m_s = 0 \leftrightarrow -1$ transitions are equal [see Eq. (S20)].

In terms of this field-dependent rate, the mean total number of detected counts accumulated in

time T_m at wait time τ is

$$N(\tau, B) = I_0 t_{\text{ro}} \frac{T_m}{t_{\text{cyc}}} \left[(1 - C) + C e^{-\Gamma_L(B)\tau} \right]. \tag{S13}$$

The minimum detectable field change δB_{min} is found by setting the SNR equal to unity for a small field perturbation δB, i.e.

$$\text{SNR} = \frac{\left|\frac{\partial N}{\partial B}\right| \delta B}{\sigma_N} \stackrel{!}{=} 1 \qquad \Longrightarrow \qquad \delta B_{\text{min}} = \frac{\sigma_N}{\left|\frac{\partial N}{\partial B}\right|}, \tag{S14}$$

where in the shot-noise-limited regime $\sigma_N \simeq \sqrt{N(\tau, B)}$. The sensitivity is defined as

$$\eta = \delta B_{\text{min}} \sqrt{T_m}\,. \tag{S15}$$

Using Eq. (S13), we obtain

$$\eta(\tau, B) = \frac{1}{C\tau e^{-\Gamma_L \tau} \left|\frac{\partial \Gamma_L}{\partial B}\right|} \sqrt{\frac{\left[(1 - C) + C e^{-\Gamma_L \tau}\right] t_{\text{cyc}}}{I_0 t_{\text{ro}}}} \stackrel{(C \ll 1)}{\approx} \frac{1}{C\tau e^{-\Gamma_L \tau} \left|\frac{\partial \Gamma_L}{\partial B}\right|} \sqrt{\frac{t_{\text{cyc}}}{I_0 t_{\text{ro}}}}\,. \tag{S16}$$

In the commonly used limit $t_{\text{cyc}} \approx \tau$, Eq. (S16) reduces to the expression $\eta(\tau, B) \approx [C\sqrt{I_0 t_{\text{ro}} \tau}\, e^{-\Gamma_L \tau} \left|\frac{\partial \Gamma_L}{\partial B}\right|]^{-1}$.

The derivative entering Eq. (S16) is

$$\frac{\partial \Gamma_L}{\partial B} = \Gamma_1 \frac{2(1 - 1/r)\lambda^2 (\omega - \omega_{\text{CR}})}{\left[(\omega - \omega_{\text{CR}})^2 + \lambda^2\right]^2} \frac{\partial \omega}{\partial B}\,. \tag{S17}$$

For simplicity one can approximate the dispersions as linear:

$$\omega_{\text{NV}} = D_{\text{NV}} + \gamma_e \mathbf{B} \cdot \mathbf{n}_{\text{NV}} \qquad \text{and} \qquad \omega_{\text{V}_\text{B}^-} = D_{\text{V}_\text{B}^-} - \gamma_e \mathbf{B} \cdot \mathbf{n}_{\text{V}_\text{B}^-} \tag{S18}$$

where $\mathbf{n}_{\text{NV}}$ and $\mathbf{n}_{\text{V}_\text{B}^-}$ are the unit vectors in the direction of the NV and V_B^- spin axes respectively, D_{NV} and $D_{\text{V}_\text{B}^-}$ are the ground state zero-field splittings, and γ_e is the electron gyromagnetic ratio.

The detuning can thus be expressed as

$$\omega(B) = \underbrace{\left(D_{\mathrm{V_B^-}} - D_{\mathrm{NV}}\right)}_{\Delta D} - \gamma_e \mathbf{B} \cdot \left(\mathbf{n}_{\mathrm{V_B^-}} + \mathbf{n}_{\mathrm{NV}}\right). \tag{S19}$$

Letting $\mathbf{B} = B\mathbf{n}_B$ one has

$$B_{\mathrm{CR}} = \frac{\Delta D}{\gamma_e \mathbf{n}_B \cdot \left(\mathbf{n}_{\mathrm{V_B^-}} + \mathbf{n}_{\mathrm{NV}}\right)} \qquad \text{and} \qquad \frac{\partial \omega}{\partial B} = -\gamma_e \mathbf{n}_B \cdot \left(\mathbf{n}_{\mathrm{V_B^-}} + \mathbf{n}_{\mathrm{NV}}\right) \equiv -\gamma_{\mathrm{CR}}. \tag{S20}$$

With $\mathbf{n}_{\mathrm{V_B^-}}$ along the z-axis, $\mathbf{n}_{\mathrm{NV}}$ at 54.7° and $\mathbf{n}_B$ at 40.2° lying in the same plane, then $\gamma_{\mathrm{CR}} \approx 1.7\gamma_e$.

To estimate the optimal τ for field sensing, consider the common regime $C \ll 1$ where $(1 - C) + Ce^{-\Gamma_L \tau} \approx 1$. Then Eq. (S16) implies

$$\eta(\tau, B) \propto \frac{\sqrt{t_{\mathrm{cyc}}}}{\tau e^{-\Gamma_L \tau}} \qquad (C \ll 1). \tag{S21}$$

If $t_{\mathrm{cyc}} \approx \tau$ this yields the familiar optimum $\tau = 1/(2\Gamma_L)$ and hence

$$\eta(B) = \frac{\sqrt{2e\Gamma_L/(I_0 t_{\mathrm{ro}})}}{C\left|\frac{\partial \Gamma_L}{\partial B}\right|}, \tag{S22}$$

evaluated at $\tau = 1/(2\Gamma_L)$. More generally, if $t_{\mathrm{cyc}} = \tau + t_{\mathrm{dead}}$ the optimum shifts slightly and can be found by minimizing Eq. (S16) numerically.

Using Eq. 3 in the main text, we find the shot noise-limited magnetic field sensitivity of the $\mathrm{V_B^-}$ $m_s = 0 \leftrightarrow -1$ ODMR dip can range from the order of 0.1 to 10 mT/$\sqrt{\mathrm{Hz}}$ depending on the linewidth and brightness, compared to the theoretical result found here on the order of 10 mT/$\sqrt{\mathrm{Hz}}$ (Fig. S13) for NV single-τ T_1-MR calculated using Eq. (S16) under the same shot-noise-limited and $t_{\mathrm{cyc}} \approx \tau$ assumptions. According to this, the NV single-τ T_1-MR approach can be comparable to direct ODMR measurement of the $\mathrm{V_B^-}$ PL.

However, this comparison implicitly assumes identical detected photon collection efficiency at the relevant wavelengths. Most commonly available Si avalanche photodiodes (APD) have a quantum efficiency that is maximized near the middle of the visible light range. Figure S14 shows the quantum efficiency as a function of wavelength for a typical APD (PicoQuant PDM series),

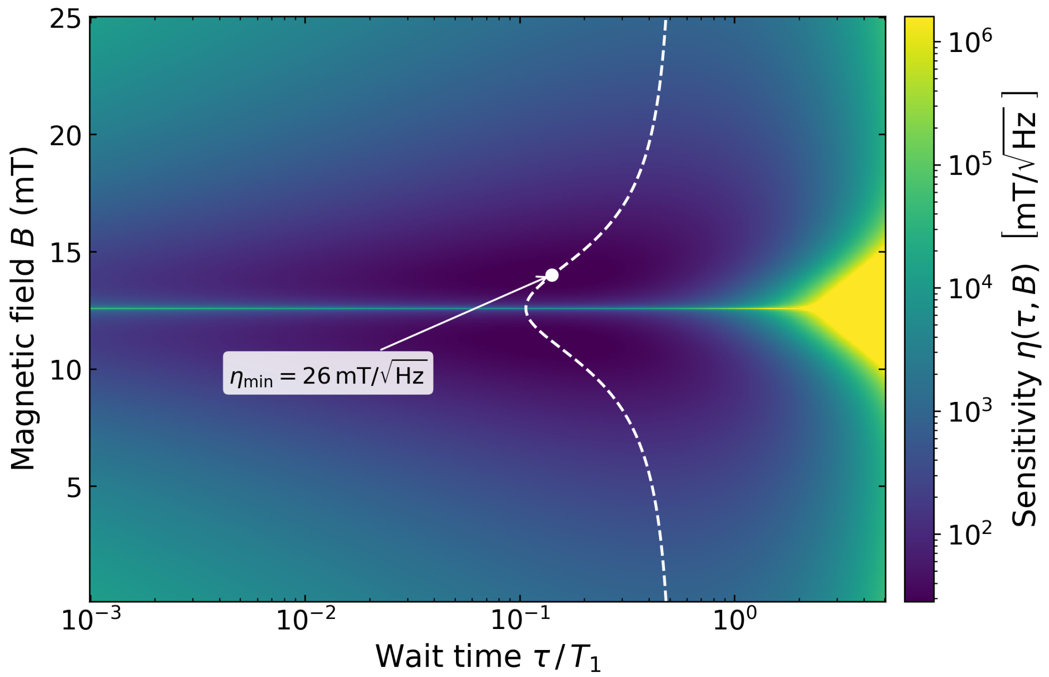

Fig. S13: **Optimization of Magnetic Field Sensitivity η of T_1-MR Measurement:** Field sensitivity η of single-τ T_1-MR measurement from Eq. (S16) as a function of the wait time τ and the applied field B at an angle of 40° from the z-axis. Plotted with $C = 0.20$, $I_0 = 500$ kcps, $t_{ro} = 500$ ns, and $r = 0.2$ assuming $t_{cyc} \approx \tau$. Dashed white line represents the approximate optimum $\tau = 1/2\Gamma_L$ in the $t_{cyc} \approx \tau$ and $C \ll 1$ limit, with the white dot showing the point of minimum sensitivity.

with a maximum near 550 nm. Since the NV PL is peaked around 700 nm compared to ~850 nm for V_B^- centers, this corresponds roughly to a factor of ~3 difference in detection efficiency for otherwise identical optical throughput. Under these assumptions, the sensitivities between these two measurement techniques become even more comparable.

While the overall efficiency of a single-τ T_1-MR measurement may be lower than direct ODMR in certain cases—such as for a sufficiently large ensemble of V_B^- centers—the advantage of the cross-relaxation approach becomes most apparent in the few-spin limit. In this regime, the relevant defects may reside in nanoscale (~1–10 nm) clusters whose PL is far below the level typically detected in confocal measurements, which collect fluorescence from a diffraction-limited volume (~500 nm lateral diameter). By contrast, scanning NV cross-relaxometry can position the NV directly above such a cluster, and the cross-relaxation contribution to the NV relaxation rate is dominated by the closest V_B^- spins and decays steeply with the NV-V_B^- separation separation as r^{-6} (Eq. (S49)). Consequently, even when the V_B^- PL is too weak for ODMR spectroscopy, the localized

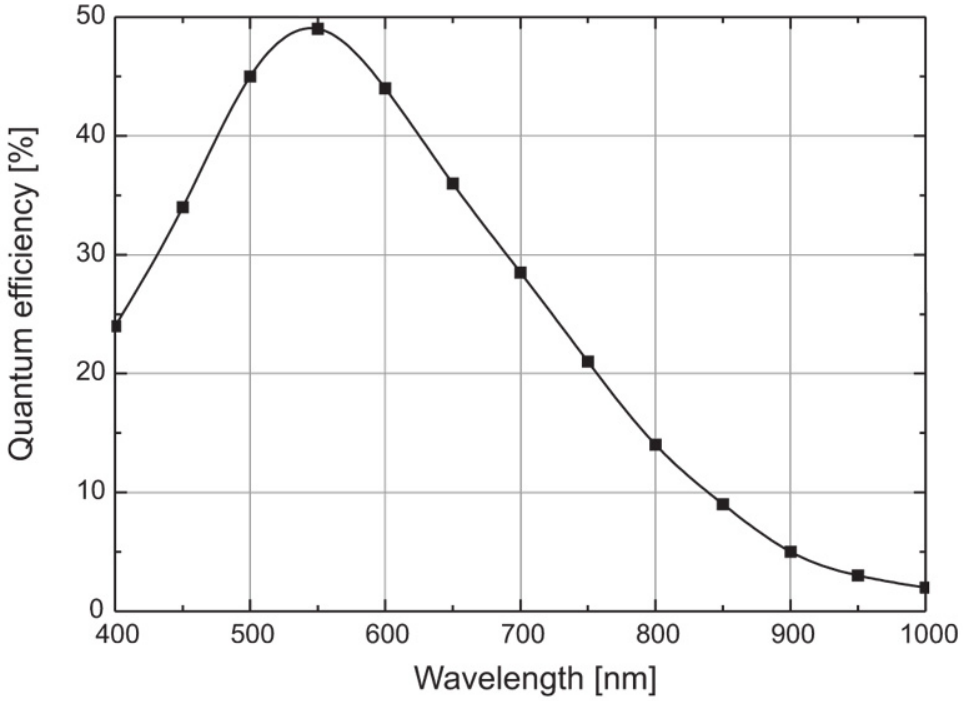

Fig. S14: **Quantum Efficiency of Typical APD:** Quantum efficiency of PicoQuant PDM Series Single Photon Avalanche Photodiode as a function of photon wavelength [figure source `https://www.picoquant.com`].

NV T_1-MR signal can remain detectable. Our technique is therefore well suited for probing the spin physics of V_B^- centers in the few-spin (or nanoscale-cluster) limit, where optical readout alone often lacks the requisite sensitivity.

To quantify the performance limits of the cross-relaxation (CR) method, we provide a quantitative estimate of the minimum detectable defect density and the feasibility of single-spin detection based on the experimental parameters derived in Supplementary Note 4. First, regarding the minimum detectable volume density, we consider the SNR in the measurement of the induced relaxation rate change. In our experiments, a V_B^- density of approximately 220 ppm yielded an induced relaxation rate change of $\Gamma_1^{CR} \approx 1.72\,\text{kHz}$ at an NV–sample distance of 11.4 nm. Under typical experimental conditions (integration time $T_m \approx 60\,\text{s}$, contrast $C \approx 0.15$), the minimum resolvable change in the relaxation rate is determined by photon shot noise to be $\delta\Gamma \approx 10$–$20\,\text{Hz}$. Assuming a linear scaling of the induced Γ_1^{CR} with density in the dilute limit (Eq. (S56)), this noise floor implies a minimum detectable V_B^- concentration of approximately 1–3 ppm.

Second, we evaluate the feasibility of detecting a single V_B^- center by considering the coupling

rate $\Gamma_{1,j}$ between the NV and a single defect at distance r. Following the derivation in Supplementary Note 4, the rate scales as $\Gamma_{1,j} \propto 1/(r^6 \cdot \Gamma_{\mathrm{tot}})$, where Γ_{tot} is the effective spin linewidth. Assuming a V_B^- linewidth of $\Gamma_{\mathrm{tot}} \approx 2\pi \times 200\,\mathrm{MHz}$ (consistent with the broad ODMR features in Fig. S8), a single V_B^- defect at $r = 10\,\mathrm{nm}$ is estimated to induce a relaxation rate change of $\Gamma_{1,j} \approx 10$–$15\,\mathrm{Hz}$. For a high-quality probe with an intrinsic relaxation rate $\Gamma_1^{\mathrm{int}} \approx 200\,\mathrm{Hz}$, this represents a ~5–8% change relative to the background rate. If we consider the narrower hyperfine features (~10s of MHz) observed in Fig. S18, the coupling increases to $\Gamma_{1,j} \approx 20$–$50\,\mathrm{Hz}$, corresponding to up to a ~10–25% change in the ideal case.

While a broader linewidth reduces the peak signal, the $1/r^6$ dependence ensures the signal increases dramatically at shorter distances. For example, at an NV–sample distance of $d = 8\,\mathrm{nm}$ with a 200 MHz linewidth, the single-spin signal rises to ~40–60 Hz, making it resolvable even against a higher noise floor ($\Gamma_1^{\mathrm{int}} \sim 1\,\mathrm{kHz}$). Notably, the estimated minimum detectable density (1–3 ppm) is physically consistent with the single-spin limit. In hBN, a 1 ppm concentration corresponds to approximately 0.1 spins/nm^3. Given the $1/r^6$ locality of the dipolar interaction, the NV sensor effectively probes a volume of $V_{\mathrm{eff}} \approx \frac{2}{3}\pi d^3$. For $d \approx 11.4\,\mathrm{nm}$ this volume contains on average less than one V_B^- defect at 1 ppm. Thus, the sensitivity threshold of the ensemble measurement naturally coincides with the regime where the signal is dominated by the single nearest defect spin. We conclude that single-spin resolution is achievable provided the standoff distance is maintained at or below ~10 nm.

Supplementary Note 4 Derivation of NV Relaxation Rate from Spin Noise in hBN

(a) Relaxation Rate Contribution from hBN Bath

The longitudinal relaxation rate $\Gamma_1 = 1/T_1$ arises from the NV spin interacting with a randomly fluctuating magnetic field perpendicular to the NV spin quantization axis. The rate depends on the spectral density of the field fluctuations at the NV spin resonance frequency ω_{NV}. We start with the autocorrelation function and compute the relaxation rate. The spectral density $J(\omega)$ is the Fourier transform of the autocorrelation function:

$$J(\omega_{\mathrm{NV}}) = \int_0^\infty \langle B_\perp(t) B_\perp(t+\tau)\rangle \, e^{-i\omega_{\mathrm{NV}}\tau} \, d\tau, \tag{S23}$$

where $B_\perp(t)$ is the amplitude of the dipolar field fluctuations perpendicular to the NV. Assuming exponential autocorrelation (see Appendix C in Ref. (*4*)):

$$\langle B_\perp(t) B_\perp(t+\tau)\rangle = \left\langle B_\perp^2 \right\rangle e^{-|\tau|/\tau_c} \tag{S24}$$

with a correlation time τ_c, then

$$J(\omega_{\mathrm{NV}}) = \left\langle B_\perp^2 \right\rangle \frac{\tau_c}{1 + (\omega_{\mathrm{NV}}\tau_c)^2} \,. \tag{S25}$$

By Fermi's golden rule,

$$\Gamma_1^{\mathrm{bath}} = \frac{3\gamma_e^2}{2\pi} J(\omega_{\mathrm{NV}}) = \frac{3\gamma_e^2 \left\langle B_\perp^2 \right\rangle}{2\pi} \frac{\tau_c}{1 + (\omega_{\mathrm{NV}}\tau_c)^2} \tag{S26}$$

where $\gamma_e = -2\pi \times 2.8\,\mathrm{MHz/G}$ is the electron gyromagnetic ratio. To obtain an expression for Γ_1^{bath}, one must find expressions for $\left\langle B_\perp^2 \right\rangle$ and τ_c.

To derive $\left\langle B_\perp^2 \right\rangle$, consider a single NV center located at the Cartesian coordinate $(0, 0, -d)$ below an infinite slab of hBN of thickness h lying from $z = 0$ to $z = +h$ containing spin-1 defects with volume density ν. The NV's quantization axis is $\hat{\mathbf{n}} = (\sin\theta, 0, \cos\theta)$. We derive the variance

of the magnetic field perpendicular to $\hat{\mathbf{n}}$, denoted $B_\perp^2$. The magnetic field at the NV location due to a bath spin $\mathbf{S}_i$ at position $\mathbf{r}_i$ is given by the dipole interaction (5):

$$\mathbf{B}_i = \frac{\mu_0 \gamma_e \hbar}{4\pi r_i^3} \left[3(\mathbf{S}_i \cdot \hat{\mathbf{r}}_i)\hat{\mathbf{r}}_i - \mathbf{S}_i\right], \tag{S27}$$

where $\mathbf{r}_i = (x_i, y_i, -d - z_i)$ is the vector from the bath spin to the NV center, $\mu_0 = 4\pi \times 10^{-7}$ H/m is the vacuum permeability and $\hbar = 1.054 \times 10^{-34}$ J s is the reduced Planck constant.

Because $B_{i,\perp}^2 = \mathbf{B}_i \cdot \mathbf{B}_i - (\hat{\mathbf{n}} \cdot \mathbf{B}_i)^2 = [\mathbf{B}_i - (\hat{\mathbf{n}} \cdot \mathbf{B}_i)\hat{\mathbf{n}}]^2$, with Eq. (S27), we can define $\left\langle B_{i,\perp}^2 \right\rangle \equiv \mathrm{Tr}\left(\rho B_{i,\perp}^2\right) \equiv (\frac{\mu_0 \gamma_e \hbar}{4\pi})^2 \mathcal{A}_i^2 r_i^{-6}$, where the maximally mixed state (infinite temperature thermal state) $\rho = \frac{1}{2s+1} \mathbb{1}_{2s+1}$ and the dipole angular factor

$$\begin{aligned}
\mathcal{A}_i^2 &= \left\langle [3(\mathbf{S}_i \cdot \hat{\mathbf{r}}_i)\hat{\mathbf{r}}_i - \mathbf{S}_i]^2 - \{\hat{\mathbf{n}} \cdot [3(\mathbf{S}_i \cdot \hat{\mathbf{r}}_i)\hat{\mathbf{r}}_i - \mathbf{S}_i]\}^2 \right\rangle && \text{(S28)}\\
&= \left\langle 3(\mathbf{S}_i \cdot \hat{\mathbf{r}}_i)^2 + \mathbf{S}_i^2 \right\rangle - \\
&\quad \left\langle 9(\hat{\mathbf{r}}_i \cdot \hat{\mathbf{n}})^2(\mathbf{S}_i \cdot \hat{\mathbf{r}}_i)^2 + (\mathbf{S}_i \cdot \hat{\mathbf{n}})^2 - 3(\hat{\mathbf{r}}_i \cdot \hat{\mathbf{n}})(\mathbf{S}_i \cdot \hat{\mathbf{r}}_i)(\mathbf{S}_i \cdot \hat{\mathbf{n}}) - 3(\hat{\mathbf{r}}_i \cdot \hat{\mathbf{n}})(\mathbf{S}_i \cdot \hat{\mathbf{n}})(\mathbf{S}_i \cdot \hat{\mathbf{r}}_i) \right\rangle && \text{(S29)}\\
&= 2s(s+1) - \left[s(s+1)(\hat{\mathbf{r}}_i \cdot \hat{\mathbf{n}})^2 + \frac{s(s+1)}{3} \right] && \text{(S30)}\\
&= \frac{s(s+1)}{3}[5 - 3(\hat{\mathbf{r}}_i \cdot \hat{\mathbf{n}})^2]. && \text{(S31)}
\end{aligned}$$

In the continuum limit one has $\mathbf{r} = (x, y, -d - z)$, and $\hat{\mathbf{r}} = (\sin\beta\cos\phi, \sin\beta\sin\phi, -\cos\beta)$, where $\cos\beta = (d+z)/r$, $r = \sqrt{\rho^2 + (d+z)^2}$, $\rho = (d+z)\tan\beta$, and $\tan\phi = y/x$. Here, $\beta \in [0, \pi/2)$ and

$\phi \in [0, 2\pi)$. The sum $\sum_i \mathcal{A}_i r_i^{-6}$ can be approximated by the integral

$$\sum_i \frac{\mathcal{A}_i^2}{r_i^6} \approx v \int_0^h \mathrm{d}z \int_0^{2\pi} \mathrm{d}\phi \int_0^{\infty} \rho \, \mathrm{d}\rho \, \frac{s(s+1)}{3} \left[5 - 3(\hat{\mathbf{r}} \cdot \hat{\mathbf{n}})^2\right] r^{-6} \tag{S32a}$$

$$= \frac{vs(s+1)}{3} \int_0^h \mathrm{d}z \int_0^{2\pi} \mathrm{d}\phi \int_0^{\pi/2} [(d+z)\tan\beta] \, \mathrm{d}[(d+z)\tan\beta] \times$$

$$\left[5 - 3(\sin\beta\cos\phi\sin\theta - \cos\beta\cos\theta)^2\right] \cos^6(\beta)(d+z)^{-6} \tag{S32b}$$

$$= \frac{vs(s+1)}{3} \int_d^{h+d} z^{-4} \mathrm{d}z \int_0^{2\pi} \mathrm{d}\phi \int_0^{\pi/2} (\tan\beta) \, \mathrm{d}(\tan\beta) \times$$

$$\left[5 - 3(\sin\beta\cos\phi\sin\theta - \cos\beta\cos\theta)^2\right] \cos^6(\beta) \tag{S32c}$$

$$= \frac{v\pi(3 - \cos^2\theta)}{2} \int_d^{h+d} \frac{\mathrm{d}z}{z^4} \tag{S32d}$$

$$= \frac{v\pi(3 - \cos^2\theta)}{6d^3} \left[1 - \left(1 + \frac{h}{d}\right)^{-3}\right]. \tag{S32e}$$

In the above, to simplify $\mathrm{d}[(d+z)\tan\beta] = (\mathrm{d}z)\tan\beta + z\,\mathrm{d}(\tan\beta)$, we used the fact $\mathrm{d}z\mathrm{d}z \equiv \mathrm{d}z \wedge \mathrm{d}z = 0$. Thus one has

$$\left\langle B_\perp^2 \right\rangle = \left(\frac{\mu_0 \gamma_e \hbar}{4\pi}\right)^2 \frac{v\pi(3 - \cos^2\theta)}{6d^3} \left[1 - \left(1 + \frac{h}{d}\right)^{-3}\right]. \tag{S33}$$

Given $\left\langle B_\perp^2 \right\rangle$, an expression for τ_c must be determined. The correlation time τ_c is defined as $\tau_c = 1/R_{\mathrm{dip}}$, where R_{dip} is the fluctuation rate of the bath spins due to intra-bath dipolar interactions.

$$\hbar R_{\mathrm{dip}} = \sqrt{\sum_{j \neq i} \left\langle H_{ij}^2 \right\rangle}, \tag{S34}$$

where the dipolar Hamiltonian is

$$H_{ij} = \frac{\mu_0 \gamma_e^2 \hbar^2}{4\pi r_{ij}^3} \left[\mathbf{S}_i \cdot \mathbf{S}_j - 3(\mathbf{S}_i \cdot \mathbf{u}_{ij})(\mathbf{S}_j \cdot \mathbf{u}_{ij})\right], \tag{S35}$$

and $\mathbf{u}_{ij} = \mathbf{r}_{ij}/r_{ij}$. For spin-1 systems in a mixed state, the expectation value of the square of the

Hamiltonian becomes

$$\left\langle H_{ij}^2 \right\rangle = \frac{8}{3}\left(\frac{\mu_0 \gamma_e^2 \hbar^2}{4\pi}\right)^2 \frac{1}{r_{ij}^6}. \tag{S36}$$

We choose a Cartesian coordinate system with xy-plane inside the slab with equal distance from top ($z = h/2$) and bottom ($z = -h/2$) surfaces. Suppose the ith site is located at z_i. For this geometry, there is translational symmetry in the slab plane, but not perpendicular to the slab plane, so $\tau_c(z_i)$ is a function of $z_i \in [-h/2, h/2]$. Due to the inversion symmetry, $\tau_c(z_i) = \tau_c(-z_i)$ so we only need to consider $z_i \in [0, h/2]$. For $z_i = 0$, a small ball of radius r_{min} containing the singularity is removed from the domain of integral, which is then partitioned into two parts, the ball of radius $h/2$ with a hollowed center and the remainder of the slab,

$$\sum_{j \neq i} \frac{1}{r_{ij}^6} \approx v \int_{-h/2}^{h/2} \mathrm{d}z \int_{\sqrt{h^2/4 - z^2}}^{\infty} \mathrm{d}\rho \frac{2\pi\rho}{(\rho^2 + z^2)^3} + v \int_0^{4\pi} \mathrm{d}\Omega \int_{r_{\text{min}}}^{h/2} \frac{r^2 \mathrm{d}r}{r^6} \tag{S37a}$$

$$= v\left[\frac{8\pi}{h^3} + \frac{4\pi}{3}\left(r_{\text{min}}^{-3} - 8h^{-3}\right)\right] \tag{S37b}$$

$$= \frac{4\pi v}{3 r_{\text{min}}^3}\left(1 - \frac{2 r_{\text{min}}^3}{h^3}\right). \tag{S37c}$$

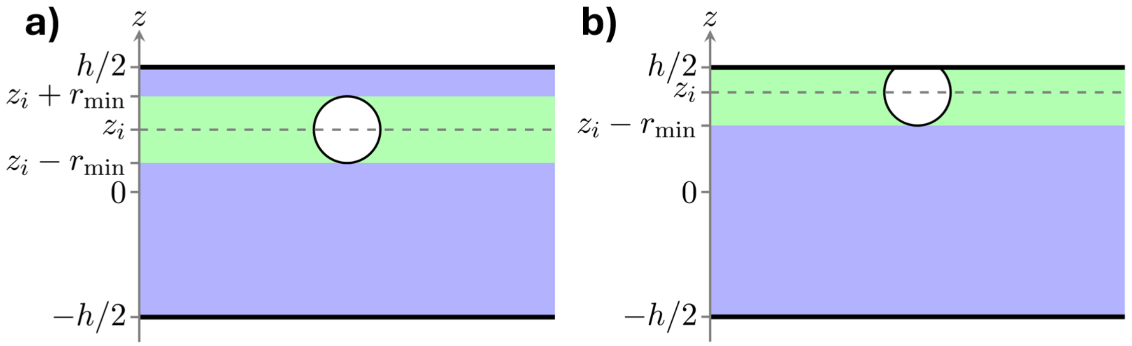

Fig. S15: **Calculation of dependence of τ_c on r_{min} given the spin depth:** (a) When $|z_i| \leq \frac{1}{2}h - r_{\text{min}}$ then the omitted volume is that of a sphere of radius r_{min}. (b) When $\frac{1}{2}h - r_{\text{min}} \leq |z_i| \leq \frac{1}{2}h$ then only that part of the sphere that intersects the slab is included.

In general, for $z_i \in [-h/2, h/2]$, we can write the integral as follows and then complete the integral by partitioning the domain according to Fig. S15: (left) two blue regions and one green region for $|z_i| \leq h/2 - r_{\text{min}}$ (this implies we assume $h > 2r_{\text{min}}$); (right) one blue region and one

green region for $h/2 - r_{\min} \le |z_i| \le h/2$.

$$\sum_{j\neq i} \frac{1}{r_{ij}^6} \approx \nu \int_{-h/2}^{h/2} \mathrm{d}z \int_{\mathrm{Re}\sqrt{r_{\min}^2-(z-z_i)^2}}^{\infty} \mathrm{d}\rho\, \frac{2\pi\rho}{[\rho^2+(z-z_i)^2]^3} \tag{S38a}$$

$$= \begin{cases} \dfrac{\pi\nu}{6}\left[8r_{\min}^{-3} - \left(\dfrac{h}{2}+|z_i|\right)^{-3} - \left(\dfrac{h}{2}-|z_i|\right)^{-3}\right], & (|z_i| \le \dfrac{1}{2}h - r_{\min}); \\ \dfrac{\pi\nu}{6}\left[4r_{\min}^{-3} - \left(\dfrac{h}{2}+|z_i|\right)^{-3} + 3r_{\min}^{-4}\left(\dfrac{h}{2}-|z_i|\right)\right], & (\dfrac{1}{2}h - r_{\min} \le |z_i| \le \dfrac{1}{2}h). \end{cases} \tag{S38b}$$

Thus the total relaxation rate of the NV due to the bath spins is (make use of Eq. (S32d) and substitute $z = d + \frac{1}{2}h - z_i$)

$$\Gamma_1^{\mathrm{bath}} = \frac{3\gamma_e^2}{2\pi} \sum_i \frac{\left\langle B_{i,\perp}^2 \right\rangle \tau_c(z_i)}{1+(\omega_{\mathrm{NV}}\tau_c(z_i))^2} \tag{S39}$$

$$= 3\gamma_e^2 \left(\frac{\mu_0\gamma_e\hbar}{4\pi}\right)^2 \frac{\nu(3-\cos^2\theta)}{4} \int_{-h/2}^{h/2} \frac{\mathrm{d}z_i}{(d+\frac{1}{2}h-z_i)^4} \frac{\tau_c(z_i)}{1+(\omega_{\mathrm{NV}}\tau_c(z_i))^2}\,. \tag{S40}$$

(b) Relaxation Rate Contribution from NV-$\mathrm{V_B^-}$ Cross-Relaxation

The cross-relaxation (CR) contribution to the relaxation rate Γ_1 occurs when the NV and a $\mathrm{V_B^-}$ center exchange energy due to near-resonant transitions, mediated by dipolar coupling. The polarization-transfer rate between NV and j^{th} $\mathrm{V_B^-}$ spin is given by Ref. (*6*)

$$\Gamma_{1,j}(\delta,\delta_j) = \left(\frac{J_0\,\mathcal{A}_j}{r_j^3}\right)^2 \frac{2\gamma}{\gamma^2+(\delta-\delta_j)^2}\,, \tag{S41}$$

where $J_0\,\mathcal{A}_j/r_j^3$ is the dipolar coupling strength to the jth $\mathrm{V_B^-}$ at distance r_j, with $\mathcal{A}_j$ capturing the angular factors; $\gamma \approx 0.5$ is the interaction-induced linewidth (from the NV's spin-echo decay); δ and δ_j are the rotating-frame detunings of the NV center and the jth $\mathrm{V_B^-}$, respectively, each drawn

independently from a Lorentzian distribution:

$$P_{\mathrm{NV}}(\delta) = \frac{\Gamma_2^{\mathrm{NV}}/\pi}{\delta^2 + (\Gamma_2^{\mathrm{NV}})^2}, \qquad P_{\mathrm{V_B^-}}(\delta_j) = \frac{\Gamma_2^{\mathrm{V_B^-}}/\pi}{(\delta_j - \Delta)^2 + (\Gamma_2^{\mathrm{V_B^-}})^2}. \tag{S42}$$

Here, $\mathcal{A}_j$ reflects the relative orientation of the two quantization axes. In our experiments, the $\mathrm{V_B^-}$ center has its quantization axis aligned along the laboratory $\hat{z}$ axis, while the NV center has a quantization axis tilted at an angle $\alpha = \frac{1}{2}\arccos\left(-\frac{1}{3}\right) \approx 54.7^\circ$ with respect to $\hat{z}$. In order for cross relaxation to happen, one needs the transitions of both NV $m_s = 0 \to +1$ and $\mathrm{V_B^-}$ $m_s = -1 \to 0$, a double-quantum process generated by $S_{\mathrm{NV}}^+ S_j^+$. Following the derivation of the angular factors in Kucsko *et al.* (7), we express the dipole–dipole interaction between the NV spin and j^{th} $\mathrm{V_B^-}$ spin in the $S^\pm$ basis as

$$H^{\mathrm{dd}} = -\left(\frac{J_0}{r_j^3}\right)^2 \left[\left(g_j^- - i\,h_j^+\right) S_{\mathrm{NV}}^+ S_j^+ \; + \; \ldots\right], \tag{S43}$$

where the coefficients are defined as:

$$g_j^\pm = \tfrac{1}{2}\left[3(\hat{r}\cdot\hat{x}_{\mathrm{NV}})(\hat{r}\cdot\hat{x}_j) - \hat{x}_{\mathrm{NV}}\cdot\hat{x}_j \;\pm\; 3(\hat{r}\cdot\hat{y}_{\mathrm{NV}})(\hat{r}\cdot\hat{y}_j) - \hat{y}_{\mathrm{NV}}\cdot\hat{y}_j\right], \tag{S44}$$

$$h_j^\pm = \tfrac{1}{2}\left[3(\hat{r}\cdot\hat{x}_{\mathrm{NV}})(\hat{r}\cdot\hat{y}_j) - \hat{x}_{\mathrm{NV}}\cdot\hat{y}_j \;\pm\; 3(\hat{r}\cdot\hat{y}_{\mathrm{NV}})(\hat{r}\cdot\hat{x}_j) - \hat{y}_{\mathrm{NV}}\cdot\hat{x}_j\right]. \tag{S45}$$

The coefficient of the relevant $S_i^+ S_j^+$ term carries the following angular dependence:

$$\begin{aligned}\mathcal{A}_j = g_j^- - i\,h_j^+ = \frac{1}{2}\Big[&(1-\cos\alpha) + 3\,\cos\phi_j\,\sin\theta_j\left(-\cos\theta_j\,\sin\alpha + \cos\alpha\,\cos\phi_j\,\sin\theta_j\right) \\ &- 3\,i\,\sin\theta_j\left(-\cos\theta_j\,\sin\alpha + (1+\cos\alpha)\,\cos\phi_j\,\sin\theta_j\right)\sin\phi_j - 3\,\sin^2\theta_j\,\sin^2\phi_j\Big].\end{aligned} \tag{S46}$$

where θ_j and ϕ_j are the polar and azimuthal angles of the vector connecting the NV and the jth $\mathrm{V_B^-}$ center (Fig. S16 a). Thus, $r_j = h/\cos\theta_j$, $\theta_j \in [0, \pi/2)$, where h is the distance from NV center to $\mathrm{V_B^-}$ plane.

Both the NV and $\mathrm{V_B^-}$ bath spectral distributions can be individually modeled as Lorentzian with linewidth Γ_2^{NV} and $\Gamma_2^{\mathrm{V_B^-}}$ and center frequencies detuned by Δ (Eq. S42). Therefore, one can express

the ensemble-averaged rate as:

$$
\begin{aligned}
\langle \Gamma_{1,j} \rangle &= \int_{-\infty}^{\infty} \mathrm{d}\delta \int_{-\infty}^{\infty} \mathrm{d}\delta_j \; P_{\mathrm{NV}}(\delta) \; P_{\mathrm{V_B^-}}(\delta_j) \; \Gamma_{1,j}(\delta, \delta_j) && \text{(S47)} \\
&= \left(\frac{J_0 \mathcal{A}_j}{r_j^3} \right)^2 \iint \frac{\mathrm{d}\delta \mathrm{d}\delta_j}{\pi^2} \frac{\Gamma_2^{\mathrm{NV}}}{\delta^2 + (\Gamma_2^{\mathrm{NV}})^2} \frac{\Gamma_2^{\mathrm{V_B^-}}}{(\delta_j - \Delta)^2 + (\Gamma_2^{\mathrm{V_B^-}})^2} \frac{2\gamma}{\gamma^2 + (\delta - \delta_j)^2} \\
&= \left(\frac{J_0 \mathcal{A}_j}{r_j^3} \right)^2 \int \frac{\mathrm{d}\delta}{\pi} \frac{\Gamma_2^{\mathrm{NV}}}{\delta^2 + (\Gamma_2^{\mathrm{NV}})^2} \frac{2(\Gamma_2^{\mathrm{V_B^-}} + \gamma)}{(\delta - \Delta)^2 + (\Gamma_2^{\mathrm{V_B^-}} + \gamma)^2} \\
&= \left(\frac{J_0 \mathcal{A}_j}{r_j^3} \right)^2 \frac{2(\gamma + \Gamma_2^{\mathrm{NV}} + \Gamma_2^{\mathrm{V_B^-}})}{(\gamma + \Gamma_2^{\mathrm{NV}} + \Gamma_2^{\mathrm{V_B^-}})^2 + \Delta^2} \, , && \text{(S48)}
\end{aligned}
$$

where $\Delta = 0$ under the cross relaxations. Therefore, the additional decay rate becomes:

$$
\langle \Gamma_{1,j} \rangle = \left(\frac{J_0 \mathcal{A}_j}{r_j^3} \right)^2 \frac{2}{\gamma + \Gamma_2^{\mathrm{NV}} + \Gamma_2^{\mathrm{V_B^-}}} \, . \qquad \text{(S49)}
$$

From this we see that the linewidth of the T_1-MR cross-relaxation feature is determined by the spectral overlap between the NV transition and the $\mathrm{V_B^-}$ transition. This overlap can be viewed as the convolution of the NV and $\mathrm{V_B^-}$ ESR lineshapes, with an additional small contribution from the interaction-induced broadening γ that appears in Eq. (S41). In practice, the dominant contribution to the full width at half-maximum (FWHM) of the single-τ T_1-MR dips in Fig. 2c,d in the main text is the inhomogeneous linewidth of the $\mathrm{V_B^-}$ ESR transition, which is set by the $\mathrm{V_B^-}$ dephasing time T_2^* (i.e., by $\Gamma_2^{\mathrm{V_B^-}}$). This $\mathrm{V_B^-}$ linewidth is significantly larger than both the intrinsic NV ESR linewidth (set by Γ_2^{NV}) and the interaction-induced broadening γ, and therefore controls the observed FWHM of the T_1-MR features.

Extensive Monte Carlo simulations were performed of a single NV center coupled to a bath of 5000 randomly distributed $\mathrm{V_B^-}$ spins at varying densities. For each density, we averaged the additional relaxation rate, $\Gamma_1^{\mathrm{CR}} = \sum_j \langle \Gamma_{1,j} \rangle$, over 100 realizations. The NV–hBN distance is set to $d = 11.4 \pm 1.5\,\mathrm{nm}$ to match the experimental conditions. Fig. 3e in the main text plots Γ_1^{CR} as a function of $\mathrm{V_B^-}$ center density, showing that Γ_1^{CR} increases with density over the simulated range. By matching the numerical results to the experimentally extracted value of $\Gamma_1^{\mathrm{CR}} = 1.72\,\mathrm{kHz}$, we infer a $\mathrm{V_B^-}$ density of approximately 220 ppm. We account for the variations in the NV-to-sample

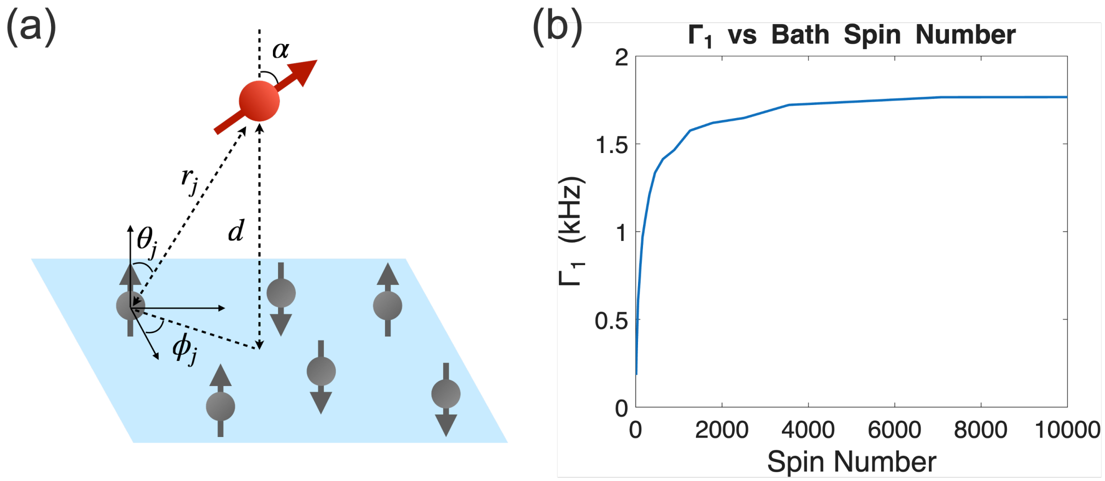

Fig. S16: **NV–V_B^- Spin Geometry:** (a) Geometry of the NV center (red spin) and V_B^- bath (grey spins), showing the tilt angle $\alpha \approx 54.7°$ of NV quantization axis and the polar (θ_j) and azimuthal (ϕ_j) angles of the NV– j^{th} V_B^- separation vector. (b) Simulated NV relaxation rate as a function of the total V_B^- number, at a V_B^- density of ∼220 ppm.

distance ($\pm 3\sigma_d = \pm 4.5$ nm), by extracting the upper and lower bounds of Γ_1^{CR} at each density (gray dashed lines). The result of a finite-size analysis of Monte Carlo simulations is shown in Fig. S16b in which the cross-relaxation rate saturates once the bath size reaches approximately 5000 spins, corresponding to NV-V_B^- separations as large as ∼50 nm. This confirms that the simulations are converged with respect to system size.

Geometric Average of $\langle \Gamma_j \rangle$ in continuum limit. Denote the (uniform) V_B^- area density σ and the area cell ΔS_j for jth V_B^- spin, so $1 = \sigma \Delta S_j$. Since $\sum_j \cdots = \sum_j \sigma \Delta S_j \cdots$,

$$\Gamma_1^{CR} = \sum_j \langle \Gamma_{1,j} \rangle \tag{S50}$$

$$= \sum_j \sigma \Delta S_j \langle \Gamma_{1,j} \rangle \tag{S51}$$

$$\approx \frac{2\sigma J_0^2}{\gamma + \Gamma_2^{NV} + \Gamma_2^{V_B^-}} \int_0^{2\pi} d\phi \int_0^{\infty} \rho d\rho \frac{\mathcal{A}^2}{r^6} \tag{S52}$$

$$= \frac{4\pi\sigma J_0^2/d^6}{\gamma + \Gamma_2^{NV} + \Gamma_2^{V_B^-}} \int_0^{2\pi} d\phi \int_0^{\pi/2} d\tan\theta \, d(d\tan\theta)$$

$$\times (-\cos\alpha + 3\cos\alpha\cos^2\theta + 3\sin\alpha\sin\phi)^2 \cos^6\theta \tag{S53}$$

$$= \frac{4\pi\sigma J_0^2/d^4}{\gamma + \Gamma_2^{NV} + \Gamma_2^{V_B^-}} \int_0^{2\pi} d\phi \int_0^{1} d\mu (-\cos\alpha + 3\cos\alpha\mu^2 + 3\sin\alpha\sin\phi)^2 \mu^3 \tag{S54}$$

$$= \frac{4\pi\sigma J_0^2/d^4}{\gamma + \Gamma_2^{NV} + \Gamma_2^{V_B^-}} \frac{3\pi}{4}(2 - \cos 2\alpha) \tag{S55}$$

$$\Gamma_1^{CR} = \frac{3\pi^2\sigma J_0^2(2-\cos 2\alpha)/d^4}{\gamma + \Gamma_2^{NV} + \Gamma_2^{V_B^-}}. \tag{S56}$$

To obtain the 3D case from the 2D result (S56), let $d \rightarrow z + d$ and $\sigma \rightarrow v\, dz$ where v is the uniform V_B^- volume density. The total contribution to the relaxation rate is then a sum of 2D sheets with differential contributions of

$$d\Gamma_1^{CR} = \frac{3\pi^2 v J_0^2 (2 - \cos 2\alpha)}{\gamma + \Gamma_2^{NV} + \Gamma_2^{V_B^-}} \frac{dz}{(z+d)^4}. \tag{S57}$$

Integrating over z, the total relaxation rate for a slab of thickness h is

$$\Gamma_1^{CR} = \frac{\pi^2 v J_0^2 (2 - \cos 2\alpha)}{\gamma + \Gamma_2^{NV} + \Gamma_2^{V_B^-}} \left(\frac{1}{d^3} - \frac{1}{(h+d)^3} \right). \tag{S58}$$

The NV-to-sample distance is of great interest as it governs spatial resolution and coupling strength. Given the constrained dependence of the NV relaxation rate on the distance to the V_B^- ensemble, the lift height dependence of the NV T_1 can be used to estimate the minimum achievable

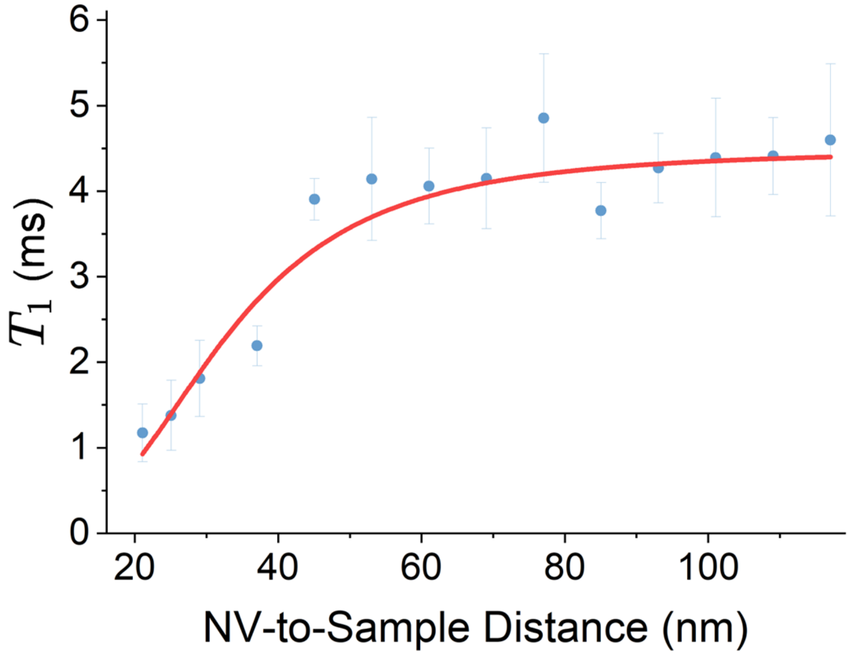

Fig. S17: **Height Dependence of NV T_1 at CR Condition:** NV T_1 measured as a function of height above the hBN_{nat} surface at the CR condition ($B = 127\,G$) as blue points. Fit to Eq. (S58) as red curve. Error bars indicate 1σ uncertainties of the fitted T_1 values.

NV-to-sample distance, and thereby put an upper bound on the distance between the NV and diamond surface. Figure S17 shows the NV probe lift height dependent T_1 above the hBN_{nat} surface shown in main manuscript Fig. 3. During the experiment, the NV (Probe #4) was initially landed onto the hBN surface with tight contact, and the magnetic field was set to achieve cross relaxation between NV and boron vacancies. Then the tip was lifted at different height using an AFM mechanism. The equation (S58) was used to fit an intrinsic relaxation time of 4.5 ms and an initial NV-to-sample distance of 21.1 ± 5.0 nm. This fitted NV-sample distance is consistent with the 19.25 ± 2.03 nm NV-sample distance obtained by scanning a CoFeB magnet (see Fig. S4). The result shows that the interaction between V_B^- and NV decays rapiadly when their distance increases, proving that this technique primarily probes the surface.

Supplementary Note 5 ODMR of V_B^- in $h^{10}B^{15}N$

The ground state Hamiltonian of the V_B^- center is (8)

$$\frac{\mathcal{H}_{gs}}{\hbar} = D_{V_B^-} S_z^2 + \gamma_e \mathbf{B} \cdot \mathbf{S} - \sum_j \gamma_n^j \mathbf{B} \cdot \mathbf{I}^j + \sum_j \mathbf{S}\mathbf{A}^j \mathbf{I}^j, \tag{S59}$$

where γ_e and γ_n^j are the electron and j^{th} nuclear gyromagnetic ratios, $\mathbf{S}$ and $\mathbf{I}$ the electron and nuclear spin operators, $D_{V_B^-} = 2\pi \times 3.48$ GHz the V_B^- zero-field splitting at room temperature, $\mathbf{B}$ the magnetic field and $\mathbf{A}^j$ the hyperfine tensor of the jth nucleus. Using the low-field approximation appropriate for our measurement regime, the hyperfine term in Eq. (S59) can be approximated as

$$\sum_j \mathbf{S}\mathbf{A}^j \mathbf{I}^j \approx S_z \sum_j \mathbf{A}_{zz}^j \mathbf{I}_z^j \tag{S60}$$

Given the ^{15}N nuclear spin $I_z^j = 1/2$, the total nuclear spin of the three neighboring ^{15}N nuclei of the V_B^- is $\sum_{j=1}^{3} I_z^j = 3/2$, yielding the projections $\sum m_I = \{-3/2, -1/2, +1/2, +3/2\}$ with splitting $\left|A_{zz}^{15\text{N}}\right|$ and respective degeneracy given by binomial coefficients 1:3:3:1. A typical ODMR spectrum is shown in Fig. S18.

Additional details of the ODMR spectra of Fig. 2b in the main text are given in Fig. S19-S21, all plotted against OOP applied field. The $\sum m_I = \pm 1/2$ peak frequencies and linewidths obtained from fitting are displayed in Fig. S19. The corresponding hyperfine splitting is plotted in Fig. S20.

Figure S21 shows the NV ODMR peak contrast, showing an expected decrease of the contrast as applied field increases. A linear fit was made to the data and the slope was used to baseline-correct the single-τ T_1 data from Figs. 3c–d of the main text. This can be justified as follows: Using the exponential decay curve described in Eq. (S1), we can consider a linear contrast $C(B) = c_0 + c_1 B$, resulting in

$$n(\tau) = n_0 \left[(1 - C(B)) + C(B) e^{-\tau/T_1} \right]. \tag{S61}$$

The single-τ signal then becomes

$$\frac{n(\tau)}{n(0)} = (1 - C(B)) + C(B)e^{-\tau/T_1} \tag{S62}$$

$$= 1 - C(B)\underbrace{\left[1 - e^{-\tau/T_1}\right]}_{\equiv\,\alpha,\ \text{const. w.r.t. } B}, \tag{S63}$$

$$= (1 - \alpha c_0) - \alpha c_1 B\,. \tag{S64}$$

Thus if the contrast is linear in B with slope c_1, then the single-τ T_1-MR signal is also linear in B with slope αc_1.

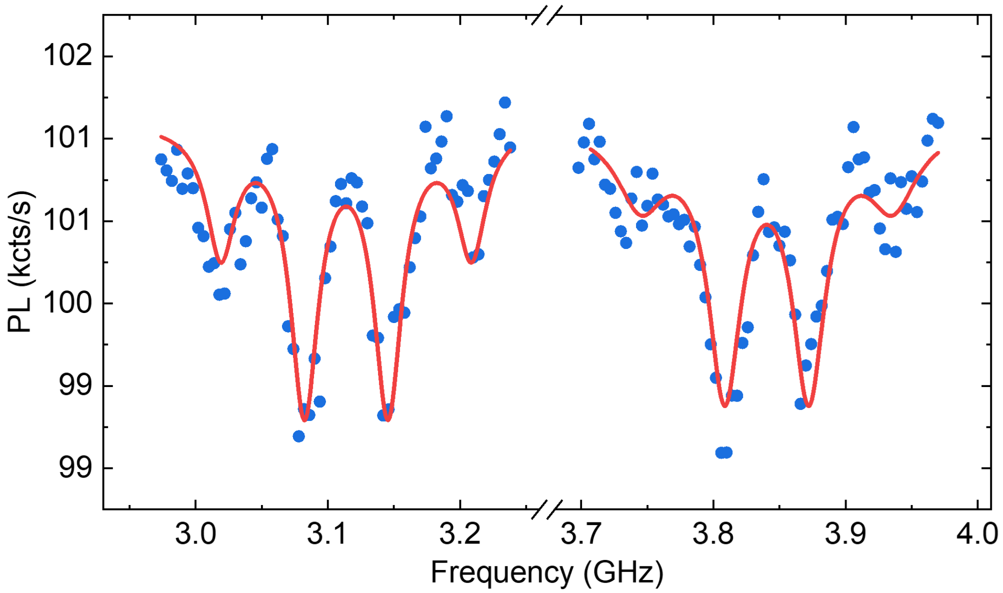

Fig. S18: **ODMR of V_B^- in $h^{10}B^{15}N$:** V_B^- ODMR spectra of both $m_s = 0 \leftrightarrow \pm1$ transitions showing hyperfine structure in $h^{10}B^{15}N$. Red curves are fits to four equally spaced Lorentzian lines. Magnetic field is applied OOP.

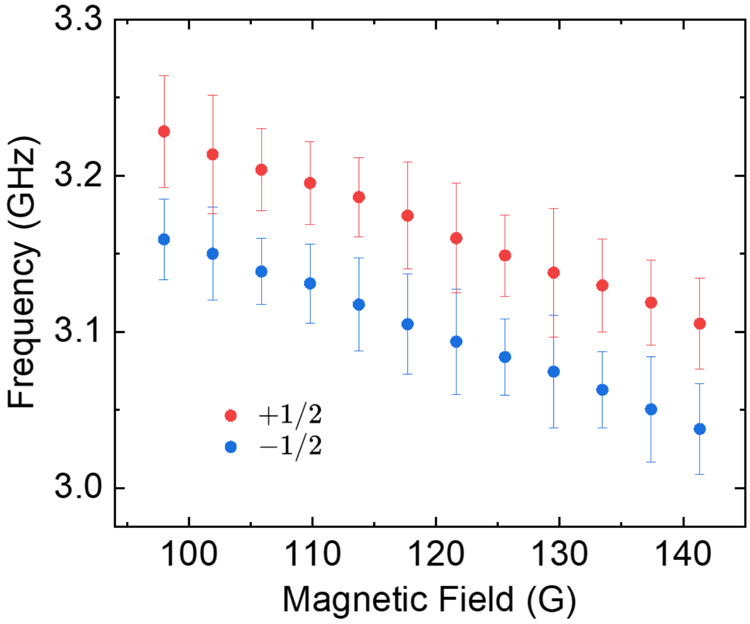

Fig. S19: **CW ODMR of V_B^- hyperfine transitions:** Center frequencies of the boron-vacancy $\sum m_I = \pm 1/2$ hyperfine structure within the $m_s = 0 \leftrightarrow -1$ transition in $h^{10}B^{15}N$. Error bars represent linewidth.

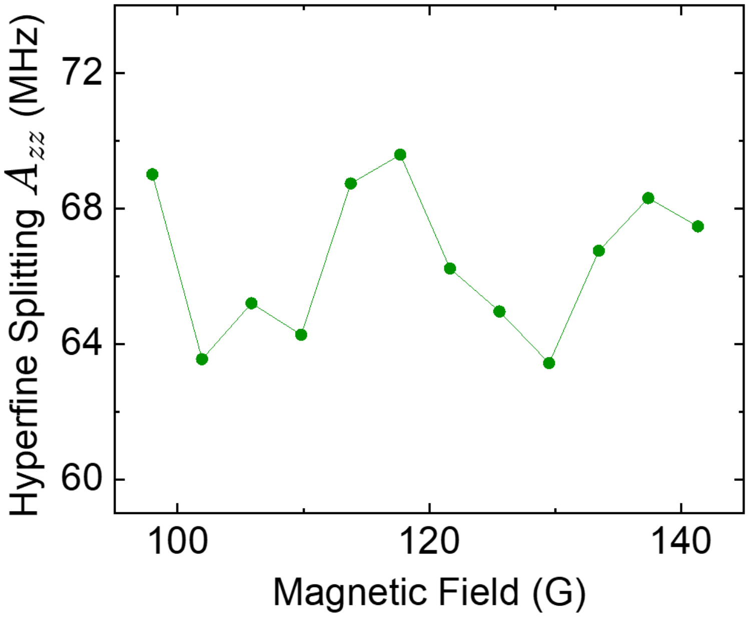

Fig. S20: V_B^- **Hyperfine Splitting:** Hyperfine splitting in $h^{10}B^{15}N$ between the $m_I = \pm 1/2$ lines shown in Fig. S19.

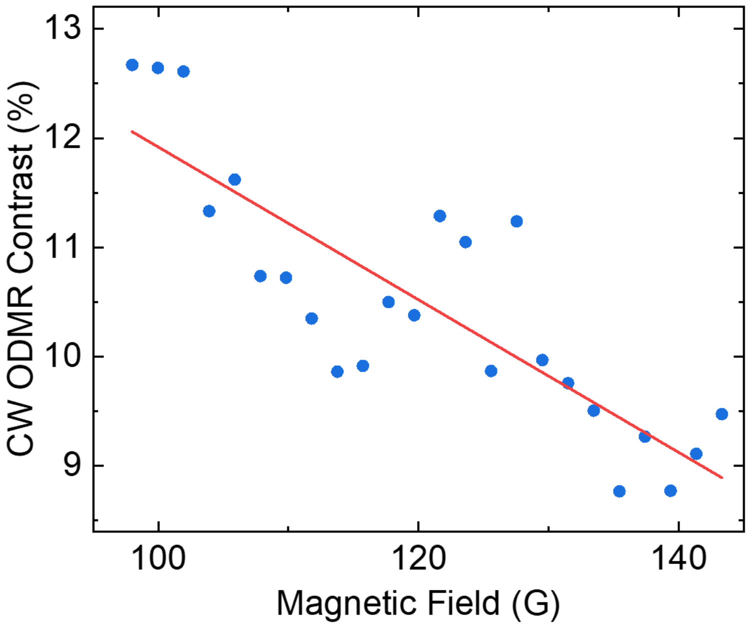

Fig. S21: **NV CW-ODMR Contrast:** CW-ODMR contrast of NV $m_s = 0 \leftrightarrow +1$ transition as a function of applied field, with linear fit in red.

Supplementary Note 6 Simulation of He Ion Irradiation in hBN/Au

To investigate the creation of V_B^- defects in hBN due to He ion irradiation, Stopping and Range of Ions in Matter (SRIM) simulations were performed to estimate the ion penetration range for the case of an hBN thickness of 90 nm vs 250 nm. We note that the choice of an Au underlayer can enhance the thickness-dependent contrast of irradiation-induced vacancy formation: Au is a heavy, high-Z material with a large elastic backscattering probability for light ions such as He^+, and therefore can produce stronger substrate-mediated backscattering than lower-Z substrates (e.g., SiO_2/Si). Figure S22 shows SRIM simulations of ion penetration. The hBN film weakly scatters incident ions relative to the high stopping power gold film. In the 90 nm case, most ions can reach the gold, which can backscatter into the weakly scattering thin hBN film allowing for a higher loss of backscattered ions at the hBN surface. In the 250 nm case, which is approaching the penetration range in hBN, most ions terminate in the hBN since the ions have lower energy once entering the gold film and are unlikely to be backscattered through the larger 250 nm hBN path. The result is that there is a more uniform termination of ions throughout the 90 nm hBN thickness as compared to the 250 nm film. Overall, this substrate-mediated backscattering effect is expected to be most pronounced when the hBN thickness is shorter than (or comparable to) the He^+ projected range, and it provides a practical mechanism for enhancing the contrast of V_B^- defect density with thickness when using an Au underlayer.

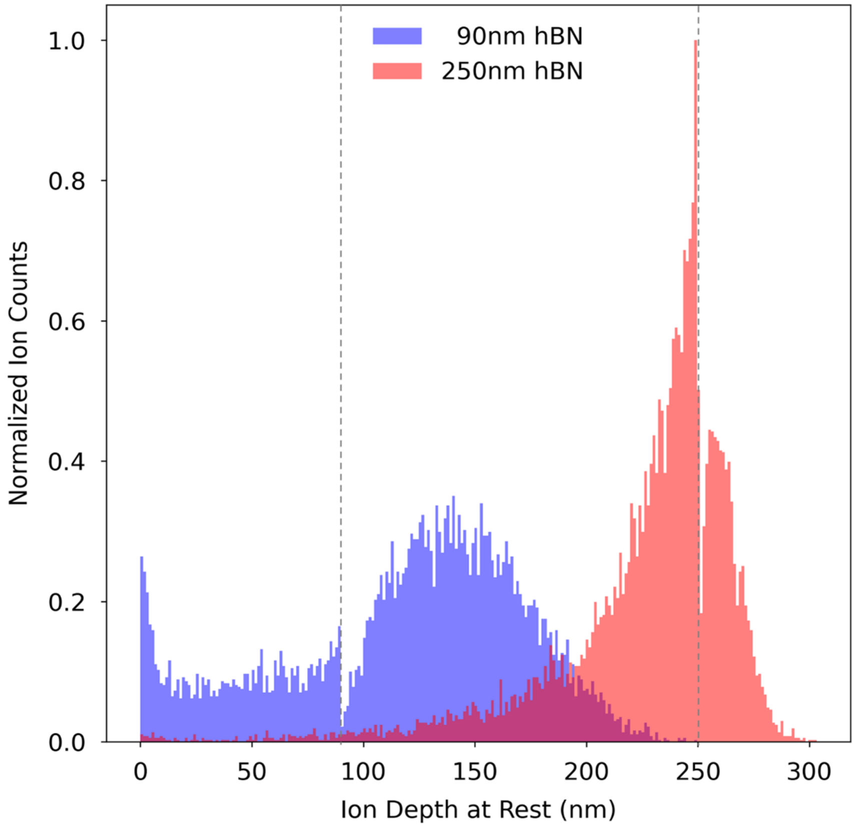

Fig. S22: **Simulated He Ion Penetration Range:** SRIM simulations of 30 keV He^{+} (10,000 ions simulated) penetration range in 90 nm and 250 nm hBN on gold. Distributions are normalized to the maximum ion count, which occurs at the hBN/Au interface of the 250 nm film. Dashed lines represent the hBN/Au interface.

Supplementary Note 7 Additional T_1-MR Measurements

As discussed in the main text, NV tips with long T_1 values provide higher sensitivity but measurements of many full PL decay curves is time-consuming, hence the use of the single-τ T_1-MR technique. Full PL decay curves for the long-T_1 NV tips (Probes #1 and #2) used for the single-τ T_1-MR measurements of $h^{10}B^{15}N$ and hBN_{nat} in Figures 2c and 2d are shown in Figs. S23a and S23b respectively, showing values of $T_1 = 2.09 \pm 0.25$ ms and 1.21 ± 0.32 ms. The T_1 was measured after landing the tip on hBN sample without applying a magnetic field.

Engaging the short T_1 probe (Probe #3) with the $h^{10}B^{15}N$ sample, a similar measurement to that of Fig. 2b in the main text consisting of 31 NV and V_B^- ODMR scans was performed with center frequencies plotted in Fig. S24. Extracting the hyperfine splitting, the hyperfine dispersions are plotted showing the four cross-relaxation conditions.

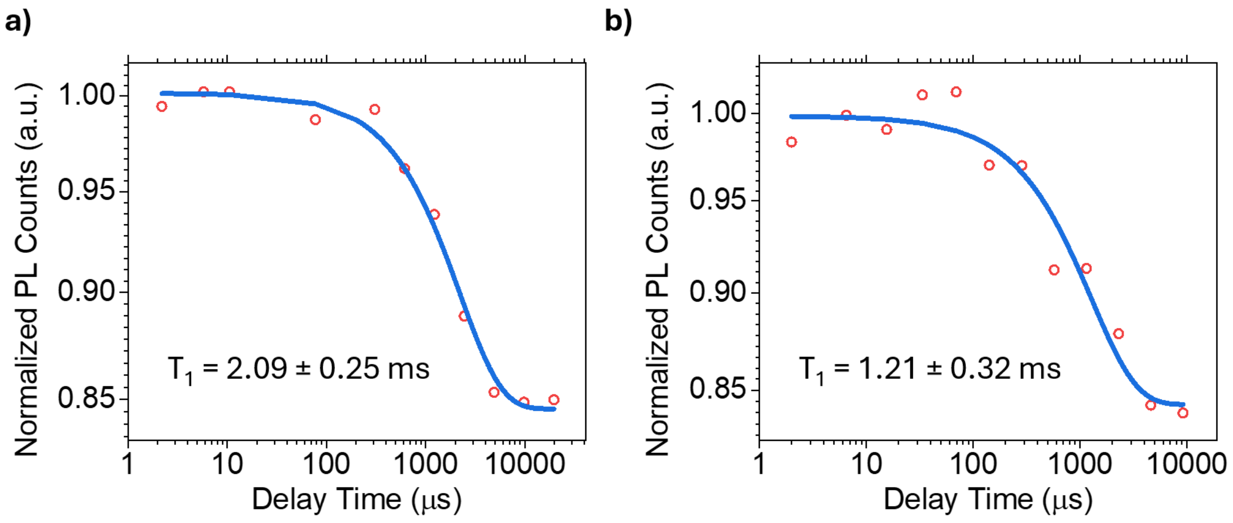

Fig. S23: **Full PL decay curves of long T_1 NV probes used for single-τ T_1-MR measurements:** PL decay of tip used for the measurements displayed in (a) Figure 2c [Probe #2] and (b) Figure 2d [Probe #1]. Blue curves are fits to a single exponential.

A fourth NV probe (Probe #4) was used to validate the single-τ cross-relaxation T_1-MR measurement of hBN_{nat} shown in Fig. 2d of the main text. The individual NV ODMR and V_B^- ODMR center frequencies are shown in Fig. S25a with the T_1-MR result shown in Fig. S25b.

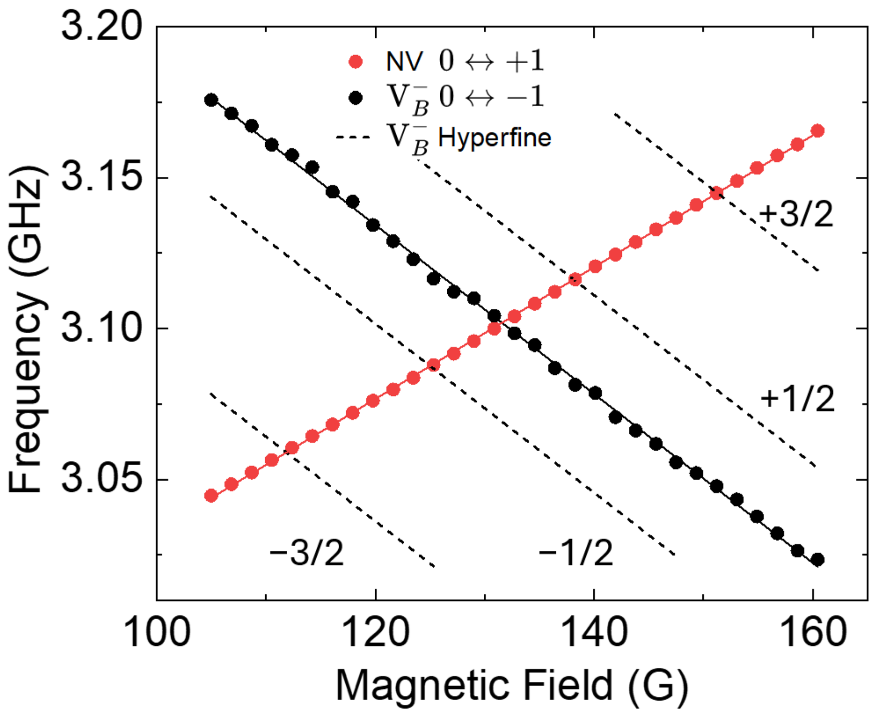

Fig. S24: **Cross-relaxation between a short-T_1 NV center and V_B^- ensembles in h^{10}B^{15}N:** Measured ODMR center frequencies for the NV $m_s = 0 \leftrightarrow +1$ transition and the V_B^- $m_s = 0 \leftrightarrow -1$ transition corresponding to plots Fig. 2e-f in the main text (Probe #3). The hyperfine structure of the V_B^- center is plotted using the measured axial hyperfine parameter $A_{zz} = 65$ MHz.

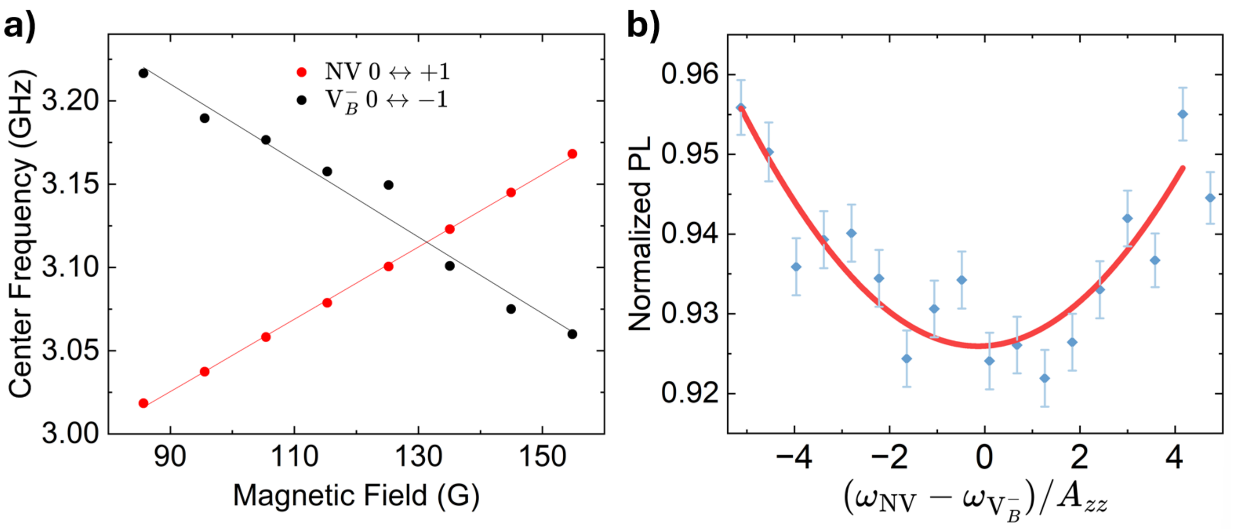

Fig. S25: **Additional T_1-MR Measurement of hBN$_{nat}$:** (a) Measured ODMR center frequencies for the NV $m_s = 0 \leftrightarrow +1$ transition and the V_B^- $m_s = 0 \leftrightarrow -1$ transition using Probe #4. (b) Single-τ T_1-MR measurement of hBN$_{nat}$ with Probe #4 and extended averaging time. Error bars represent normalized shot noise level.

Supplementary Note 8 Gate-tunable Modulation of Near-Surface V_B^- Density

This section is to demonstrate a unique application of the cross-relaxometry technique we developed. A key open question in the hBN defect community is the extent to which electrostatic gating can modulate the surface density and charge state of boron-vacancy defects. Previous optical studies have reported mixed conclusions. One work (*9*) observed essentially no gate dependence of the V_B^- density in relatively thick hBN flakes (~80 nm), while another reported only a few-percent modulation of V_B^- related photoluminescence in thinner samples under gating (*10*). These controversial results suggest the gate tuning is more effective for thinner hBN flakes. Motivated by these reports, we hypothesize that electrostatic gating primarily modifies the V_B charge-state population near the hBN surface via band bending, analogous to the well-established $NV^- \leftrightarrow NV^0$ charge-state conversion at diamond surfaces (*11*). In this scenario, any gate-induced change is spatially localized within a shallow surface region and is therefore strongly diluted in PL measurements that integrate emission from the entire flake thickness, particularly for thick hBN, which explains the inconsistency of previous two reports.

By contrast, scanning NV cross-relaxometry provides a direct and selective probe of the surface-proximal V_B^- spin density that couples to the NV center at the cross-relaxation condition. In the measurements presented in (Fig. S26), we apply a gate voltage while monitoring the NV longitudinal relaxation rate T_1^{-1} at the NV–V_B^- cross-relaxation field. We observe a clear and reproducible gate-dependent change in the cross-relaxation-induced relaxation rate, corresponding to an approximately 30% modulation of the near-surface V_B^- density.

This relatively large modulation contrasts with the few-percent gate dependences inferred from bulk-averaged PL in previous studies, and is consistent with a scenario in which the gate primarily recharges V_B^- and V_B^0 in a surface-proximal layer while deeper defects remain comparatively insensitive. These results suggest a surface band-bending picture and demonstrate that scanning NV cross-relaxometry enables quantitative, non-invasive, and charge-state-selective detection of gate-tunable spin-defect densities in two-dimensional materials, which is difficult to achieve using existing optical approaches.

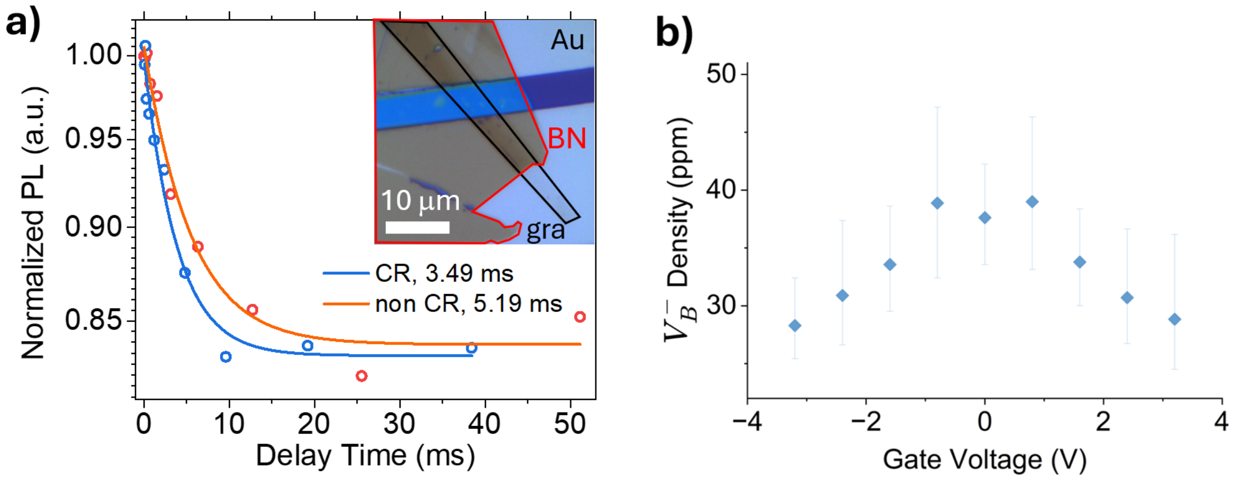

Fig. S26: **Gate-Dependence of V_B^- Surface Density:** (a) NV longitudinal spin relaxation (T_1) curve measured with Probe #4 positioned on the gated device. The extracted relaxation times are $T_1 = 5.19 \pm 0.73$ ms away from the cross-relaxation condition and $T_1 = 3.49 \pm 0.35$ ms at the NV–V_B^- cross-relaxation field. Inset: Optical micrograph of the device, consisting of a ~15 nm-thick $h^{10}B^{15}N$ flake on a gold bottom gate, with a monolayer graphene flake transferred on top of the $h^{10}B^{15}N$ and serving as the top gate. (b) V_B^- volume density calculated from the NV relaxation rate at cross-relaxation condition as a function of the applied gate voltage. The data was collected using Probe #4. Error bars represent propagated uncertainty from fitted T_1 values.

Supplementary Note 9 Additional Imaging of hBN_{nat} Sample

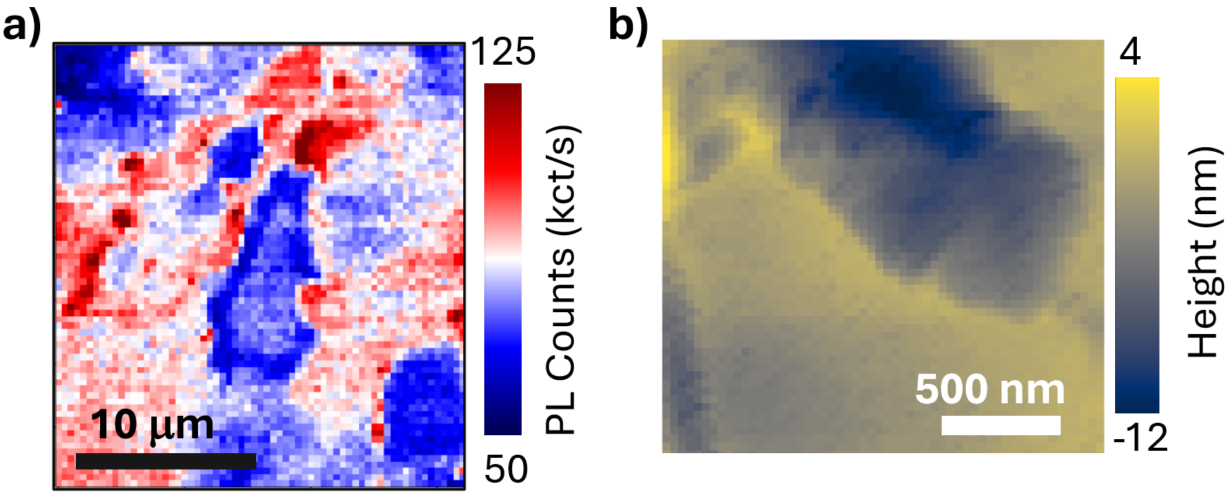

Fig. S27: **Gate-Dependence of V_B^- Surface Density:** Additional imaging of the CVD-grown hBN_{nat} sample shown in Fig. 3. (a) Confocal PL intensity map of the sample. (b) High-resolution AFM topography of the top-right region corresponding to the area imaged in Fig. 3f.

References

1. Engel-Herbert, R. & Hesjedal, T. Calculation of the magnetic stray field of a uniaxial magnetic domain. *Journal of Applied Physics* **97**, 074504 (2005). URL https://doi.org/10.1063/1.1883308. https://pubs.aip.org/aip/jap/article-pdf/doi/10.1063/1.1883308/10657531/074504_1_online.pdf.

2. Hingant, T. *et al.* Measuring the magnetic moment density in patterned ultrathin ferromagnets with submicrometer resolution. *Phys. Rev. Appl.* **4**, 014003 (2015). URL https://link.aps.org/doi/10.1103/PhysRevApplied.4.014003.

3. Xu, Z. *et al.* Minimizing sensor-sample distances in scanning nitrogen-vacancy magnetometry. *ACS Nano* **19**, 8255–8265 (2025). URL https://doi.org/10.1021/acsnano.4c18460. PMID: 39983234, https://doi.org/10.1021/acsnano.4c18460.

4. Slichter, C. P. *Principles of Magnetic Resonance*. Springer Series in Solid-State Sciences (Springer, Berlin, Heidelberg, 1990), 3 edn.

5. Tetienne, J.-P. *et al.* Spin relaxometry of single nitrogen-vacancy defects in diamond nanocrystals for magnetic noise sensing. *Phys. Rev. B* **87**, 235436 (2013). URL https://link.aps.org/doi/10.1103/PhysRevB.87.235436.

6. Zu, C. *et al.* Emergent hydrodynamics in a strongly interacting dipolar spin ensemble. *Nature* **597**, 45–50 (2021).

7. Kucsko, G. *et al.* Critical thermalization of a disordered dipolar spin system in diamond. *Physical review letters* **121**, 023601 (2018).

8. Gong, R. *et al.* Isotope engineering for spin defects in van der waals materials. *Nat. Comm.* **15**, 104 (2024).

9. Gale, A. *et al.* Manipulating the charge state of spin defects in hexagonal boron nitride. *Nano Letters* **23**, 6141–6147 (2023).

10. Fraunié, J. *et al.* Charge state tuning of spin defects in hexagonal boron nitride. *Nano Letters* **25**, 5836–5842 (2025).

11. Grotz, B. *et al.* Charge state manipulation of qubits in diamond. *Nature communications* **3**, 729 (2012).
